# Characterization of the olive endophytic community in genotypes displaying a contrasting response to *Xylella fastidiosa*

**DOI:** 10.1186/s12870-024-04980-2

**Published:** 2024-04-25

**Authors:** Marzia Vergine, Federico Vita, Paola Casati, Alessandro Passera, Luigi Ricciardi, Stefano Pavan, Alessio Aprile, Erika Sabella, Luigi De Bellis, Andrea Luvisi

**Affiliations:** 1 Department of Biological and Environmental Sciences and Technologies, University of Salento, Lecce, Italy; 2 Department of Biology, University of Bari “Aldo Moro”, Bari, Italy; 3 Department of Agricultural and Environmental Sciences, Production, Landscape, Agroenergy, University of Milan, Milano, Italy; 4 Department of Soil, Plant and Food Science, University of Bari “Aldo Moro”, Bari, Italy

**Keywords:** Ecological function, Metabarcoding, Olive cultivars, *Phaffia*, *Rhodotorula*

## Abstract

**Background:**

Endophytes mediate the interactions between plants and other microorganisms, and the functional aspects of interactions between endophytes and their host that support plant-growth promotion and tolerance to stresses signify the ecological relevance of the endosphere microbiome. In this work, we studied the bacterial and fungal endophytic communities of olive tree (*Olea europaea* L.) asymptomatic or low symptomatic genotypes sampled in groves heavily compromised by *Xylella fastidiosa* subsp. *pauca*, aiming to characterize microbiota in genotypes displaying differential response to the pathogen.

**Results:**

The relationships between bacterial and fungal genera were analyzed both separately and together, in order to investigate the intricate correlations between the identified Operational Taxonomic Units (OTUs). Results suggested a dominant role of the fungal endophytic community compared to the bacterial one, and highlighted specific microbial taxa only associated with asymptomatic or low symptomatic genotypes. In addition, they indicated the occurrence of well-adapted genetic resources surviving after years of pathogen pressure in association with microorganisms such as *Burkholderia*, *Quambalaria*, *Phaffia* and *Rhodotorula*.

**Conclusions:**

This is the first study to overview endophytic communities associated with several putatively resistant olive genotypes in areas under high *X. fastidiosa* inoculum pressure. Identifying these negatively correlated genera can offer valuable insights into the potential antagonistic microbial resources and their possible development as biocontrol agents.

**Supplementary Information:**

The online version contains supplementary material available at 10.1186/s12870-024-04980-2.

## Background

Endophytes are an endosymbiotic group of microorganisms (bacteria and fungi) that colonize inter-and/or intracellular sites of plants [1], whose study largely benefited from recent advances in sequencing technologies [2]. Their distribution is ubiquitous and, almost without exception, they display interactions with their hosts, involving mutualism, antagonism, or parasitism [3].

Bacterial and fungal endophytes act in different manners to improve plant health [4]. This goal is achieved in different ways according to symbiotic relationships, enhancing host growth, improving the plant’s fitness to biotic and abiotic stress, and improving nutrient gain [5]. Indeed, specific microbial taxa can protect plants by competing with phytopathogens for the same ecological niche through the production of inhibitors or the induction of systemic resistance in the host [6]. In addition, bacterial endophytes were shown to prevent disease development through *de novo* synthesis of compounds and antimicrobial metabolites [7].

From its first detection at the end of 2013 in the Salento peninsula (Apulia region, southern Italy), *Xylella fastidiosa* represents an emerging threat in Europe, associated with a previously unknown disease on olive trees (*Olea europaea* L.) which causes leaf scorch, a rapid decline and the death of trees (the so-called “olive quick decline syndrome”, OQDS) [8]. The symptoms are particularly severe on large old plants of the most susceptible cultivars Cellina di Nardò and Ogliarola [9], the most widespread in the whole area. Moreover, the further unexpected detections in France and Spain allow it as an expanding global pathogen [10], which could reshape not only agriculture but the landscape and human relations with plants [11]. Unfortunately, no treatment is currently available to contrast the bacterium [12].

The research on the olive microbiome delivered mounting evidence that specific microbial taxa co-occurring with pathogens may impact the disease process [13]. For example, some non-pathogenic bacteria overlapping with *Pseudomonas savastanoi* pv. *savastanoi* (*Psv*) were shown to increase resistance to olive knot disease [14]. However, the role of the olive xylem microbiota in pathogenesis has been poorly investigated [15], despite the modification or attenuation of the diversity and composition of xylem microbial communities might result in different responses to vascular pathogens [16, 17]. Understanding the close relationships between xylem-inhabiting microorganisms and *Xfp* might help researchers to identify microbial players associated with resilience to the infection, which might enable to restore landscape and agricultural settings destroyed by *Xfp* [11]. Such microbiota could also be transmitted to the progeny in olive trees vegetatively propagated, thus representing a way to increase the possible presence of beneficial symbionts within the plant [18]. Furthermore, other research explored the involvement of microbial endophytes residing in the sapwood of Apulian olive cultivars that might be a promising control strategy for xylem-colonizing pathogens such as *Xfp* and in the expression of resistance characteristics against OQDS [19]. More specifically, *Bacillus subtilis*, *Bacillus pumilus*, and *Paenibacillus rigui* were also able to secrete inhibitory substances in cell-free suspensions [20]. These findings suggest that further studies should be conducted to gain deeper insights into the role of the endophytic microbiome at the level of olive cultivars tolerance or resistance to *Xfp* infections.

For this reason, the main aims of this work were: (i) assess the leaf petiole endophytic profile of several selected genotypes by metabarcoding analysis, (ii) understand whether disease resistance is associated with a reference profile or with peculiar taxa and (iii) find relationships between genetic clusters of selected genotypes and their microbiome profile. The results might represent a step forward in understanding the plant-microbiota relationship, whose high diversity can be beneficial in contrasting the effect of *Xfp* infection and in devising a quick method to identify potentially resistant genotypes to this devastating pathogen.

## Results

### Composition of bacterial and fungal communities

**Fig. 1:**
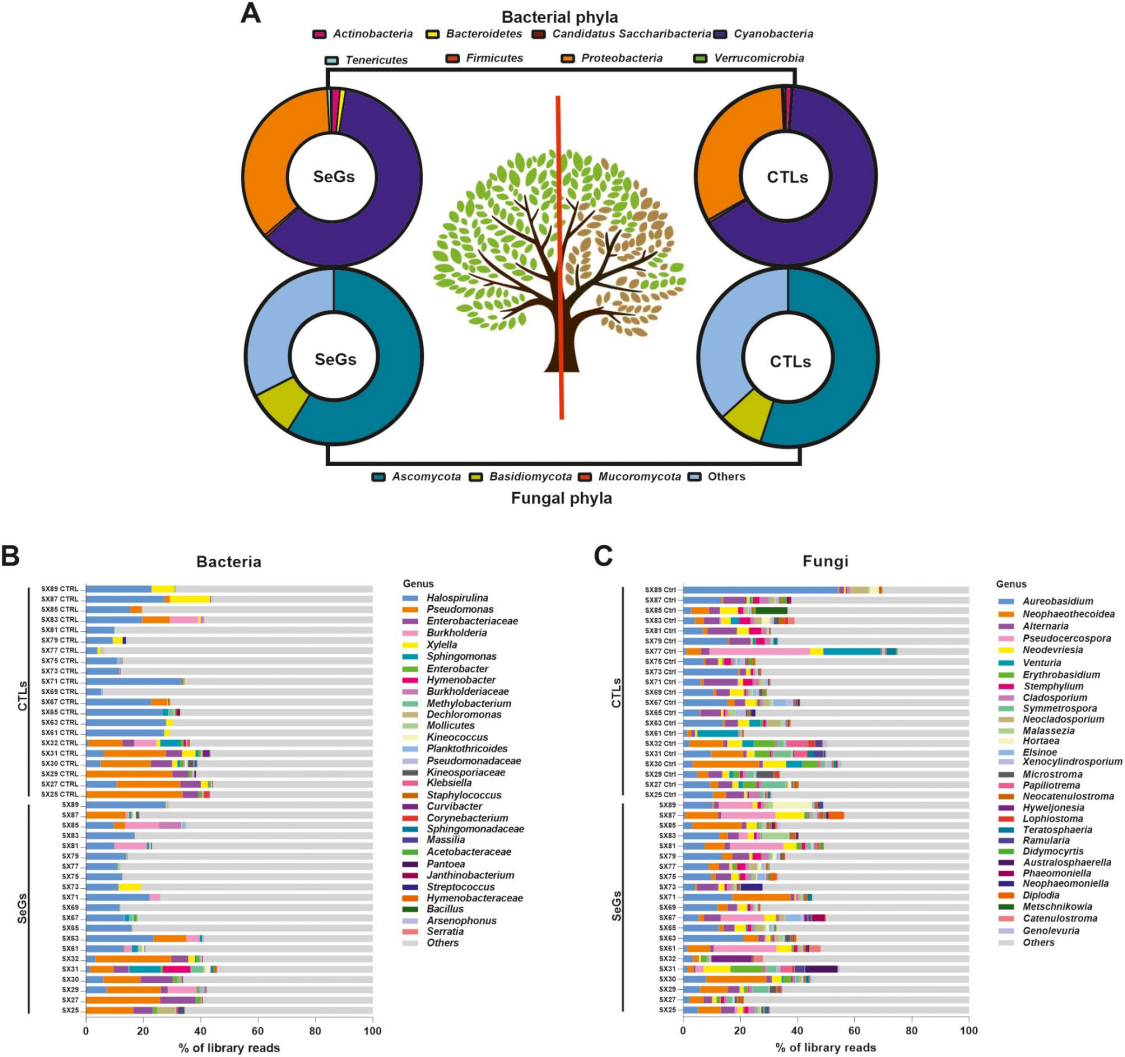
(**A**) Relative abundance of bacterial and fungal OTUs, both selected genotypes (SeGs, asymptomatic or low symptomatic trees, left) and control genotypes (CTLs, symptomatic trees, right) at taxonomic level phyla. (**B**, **C**) Taxonomy map related to control genotypes (CTLs) and selected genotypes (SeGs) microbiome. The graphs show the 30 most abundant bacterial (**B**) and fungal (**C**) OTUs at taxonomic level genus (or closely related reliable taxonomic rank), according to their best identification

The taxonomic assignment of prokaryotic OTUs revealed nine phyla, 16 classes, 40 orders, 57 families, and 72 genera belonging to the Eubacteria kingdom, with no Archaea identification. Cyanobacteria were the predominant phylum, with an average relative abundance per sample of 60.61%, followed by Proteobacteria (35.43%), Actinobacteria (1.44%), Bacteroidetes (1.05%), Tenericutes (0.65%), Firmicutes (0.54%), Verrucomicrobia (0.23%) and ‘*Candidatus* Saccharibacteria’ (0.043%) (Fig. 1A). The most abundant families were Spirulinaceae (52.25%) and Pseudomonadaceae (18.12%).

The taxonomic assignment of eukaryotic OTUs revealed four phyla, 21 classes, 64 orders, 109 families, and 172 genera. Ascomycota represented the predominant phylum with an average relative abundance per sample of 57.31%, followed by Basidiomycota (8.73%) and Mucoromycota (0.028%). The presence of unidentified fungi was also found (33.93%) (Fig. 1A). The most abundant families were Teratosphaeriaceae (13.48%) and Saccotheciaceae (12.83%).

A graph at the genus level was generated by comparing the bacterial and fungal communities of SeGs against CTLs (Fig. 1B-C). In both cases, the most abundant prokaryotic OTUs were *Halospirulina* and *Pseudomonas* (Fig. 1B, Tab. S1). As for fungal OTUs, the communities identified in both conditions were very similar, and displayed prevalence of *Aureobasidium* and *Neophaeothecoidea* (Fig. 1C, Tab. S2).

### Microbial diversity and variation in microbial community

For each taxonomic level, the *α*-diversity and *β-*diversity metrics were calculated. *Α*-diversity made it possible to evaluate wealth (OTU number) and uniformity (the relative abundance of OTUs) in the samples, while *β*-diversity allowed for an estimate of the distance or the degree of diversity between samples from SeGs and CTLs.

Phylogenetic diversity (*α*-diversity), estimated using the Chao1, Shannon, Simpson, and Fischer indexes, and the number of observed OTUs, was not reduced in CTLs compared to SeGs (Tab. S3A, B).

Despite that, differences in richness were generally attributed to fungal samples compared to bacterial ones because of higher *α*-diversity values.

**Fig. 2:**
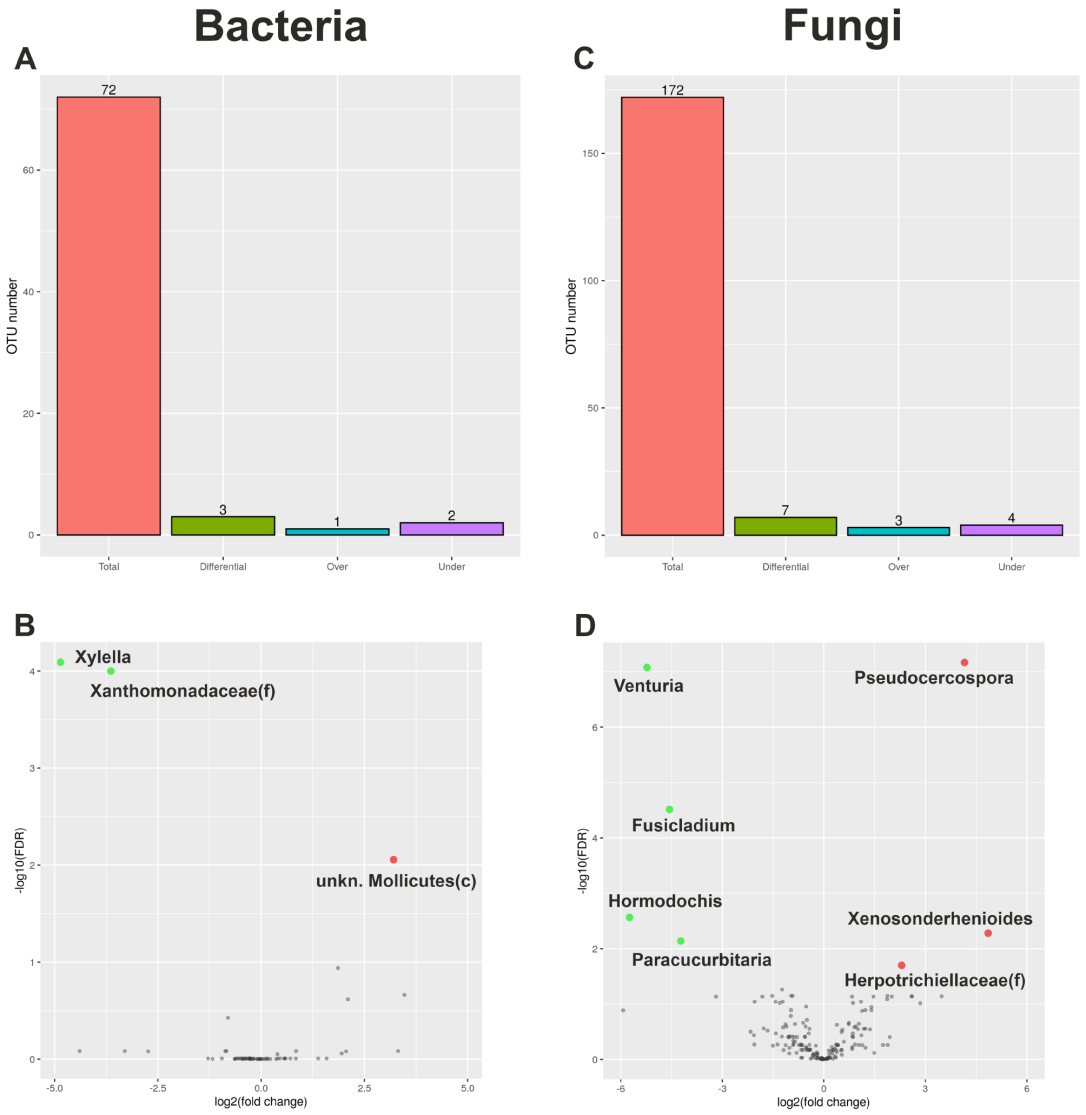
Results from GAIA pipeline. Information referring to the bacterial (**A**, **B**) and fungal (**C**, **D**) genus taxonomic level (or closely related reliable taxonomic rank). (**A**, **C**) Barplots report the total number of identified genus, the total number of differentially represented genera (Diff), and the number of over- and under-represented genera (FDR ≤ 0.05). (**B**, **D**) Volcano plots show the relationship between the fold-change (on the X-axis) and the significance of the differential abundance test (Y-axis). Black dots represent the genera that are not significantly differentially, while red and green dots are the genera that are significantly over- and under-regulated, respectively

*Β*-diversity data for bacteria and fungi (Fig. 2) at the taxonomic level genus, indicates the total number of identified genera, the number of differentially represented genera, the number of under-represented and over-represented genera. As shown in Figs. 2A and 72 bacterial genera were identified in the whole dataset. Compared to the CTLs, SeGs displayed one over-represented genus (belonging to the Mollicutes) and two under-represented genera (*Xylella* and an unidentified genus of the Xanthomonadaceae family) (Fig. 2B).

As reported in Fig. 2C, 172 fungal genera were identified, including three over-represented and four under-represented with respect to CTLs. The three over-represented genera were *Pseudocercospora*, *Xenosonderhenioides*, and an unidentified genus of the Herpotrichiellaceae family; the four under-represented genera were *Venturia*, *Fusicladium, Hormodochis*, and *Paracucurbitaria* (Fig. 2D).

**Fig. 3:**
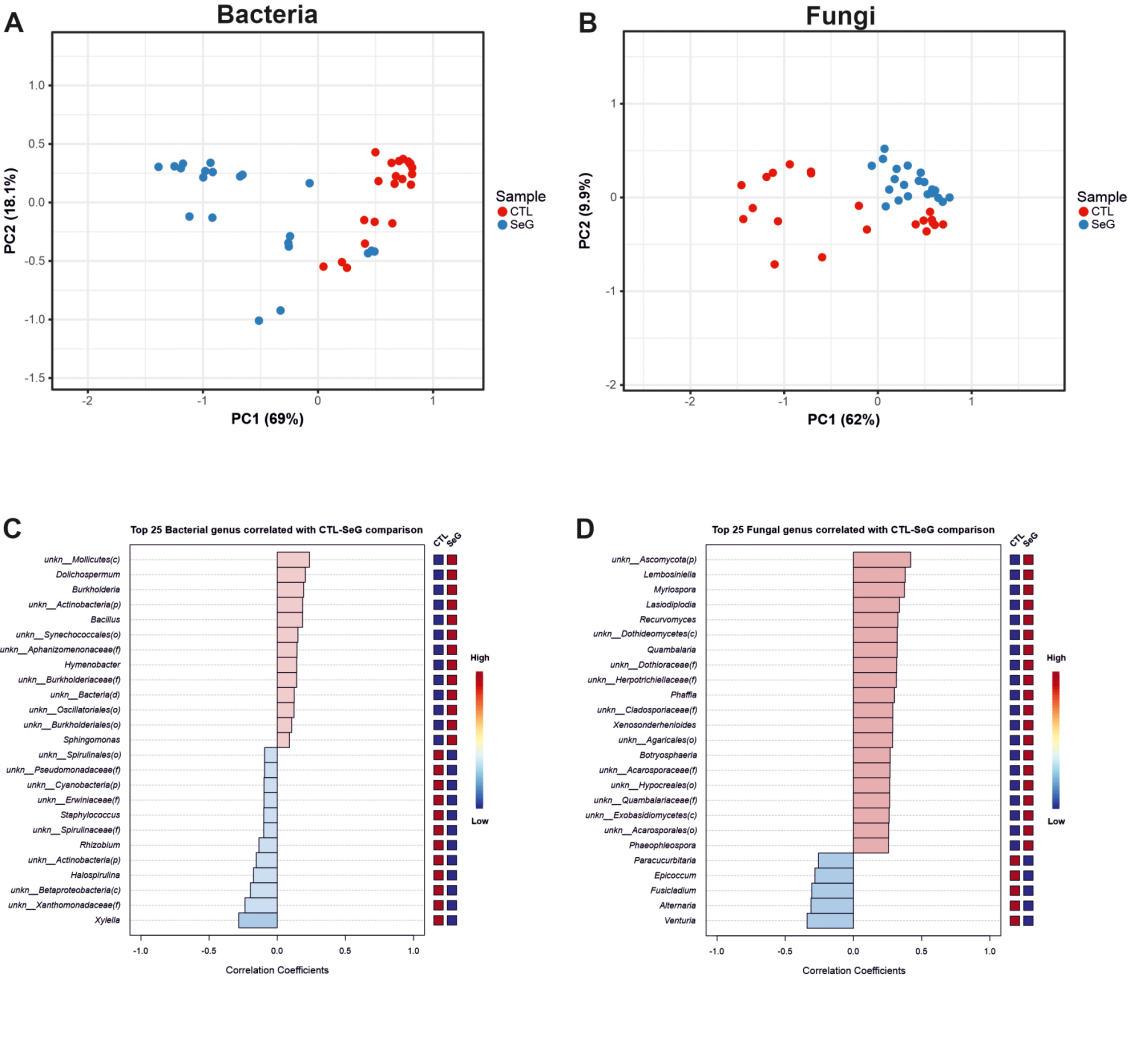
Principal Component Analysis (PCA) based on the Bray-Curtis distance matrix of bacterial (**A**) and fungal (**B**) genus taxonomic level (or closely related reliable taxonomic rank). Red points indicate the Control plants (CTLs) group samples and blue points the Selected genotypes (SeGs) group samples. (**C**-**D**) Correlation patterns of the top 25 features correlated with the bacterial (**C**) and fungal (**D**) taxa of interest. The 25 genera (or closely related reliable taxonomic rank) were ranked by their correlation. The blue color represents negative correlations, whereas the red represents positive correlations. The mini heatmap on the plot’s right side shows the two groups’ high or low abundance

Read counts at “genus” taxonomic level were also used to carry out principal component analysis (PCA) according to the Bray-Curtis distance matrix for bacterial and fungal data (Fig. 3) (Tab. S3, S4). Concerning bacterial data, the first two components (PC1 and PC2) accounted for 69% and 18.1% of the total variance, respectively (Fig. 3A). CTLs formed a homogenous group, whereas SeGs were more widely distributed, mostly according to PC1. As for fungal data, PC1 and PC2 explained 62% and 9.9% of the total variance, respectively. SeGs formed a more homogenous group with respect to CTLs.

### Community profiling and correlation analysis using MicrobiomeAnalyst

Data were also analyzed using a combination of pattern correlation and heat map analyses, with the aim to identify highly abundant bacterial and fungal genera differentially occurring in sample groups. For this purpose, OTU counts were rarefied using the minimum sample size value which corresponded to 17,537 and 56,799 reads for bacteria and fungi, respectively. As a result, rarefaction curves (Fig. S1 and S2) indicated that a sufficient sequencing depth was achieved for all the analyzed samples.

Strong relationships were identified among samples and some specific taxa. A positive correlation between SeGs and the bacterial genera *Burkholderia*, *Dolicospermun*, *Bacillus*, *Hymenobacter*, and *Sphingomonas* was found, whereas a negative correlation was highlighted between SeGs and the genus *Xylella* (Fig. 3C). As for fungal taxa, positive correlation was found between SeGs and *Lembosiella*, *Myriospora*, *Recurvomyces*, *Quambalaria*, *Phaffia*, and *Phaephleospora*, whereas negative correlation was found between SeGs and *Venturia*, *Alternaria*, *Fusicladium*, *Epicoccum* and *Paracurbitaria* (Fig. 3D).

Heat map analysis and clustering of the highly represented bacterial genera and fungal families (Fig. S3, S4) showed as taxa form separated clusters according to their abundances. Bacterial data at the genus level indicates as five distinctive clusters were formed according to Ward’s method, one of which grouped most of the highly represented taxa (Fig. S3), with samples that were also grouped in two main clusters. Looking at fungal data, the heatmap reported in Figure S4 based on family rank depicted a more complex scenario, with OTUs classified in four main clusters, whereas samples were grouped in three main groups.

**Fig. 4:**
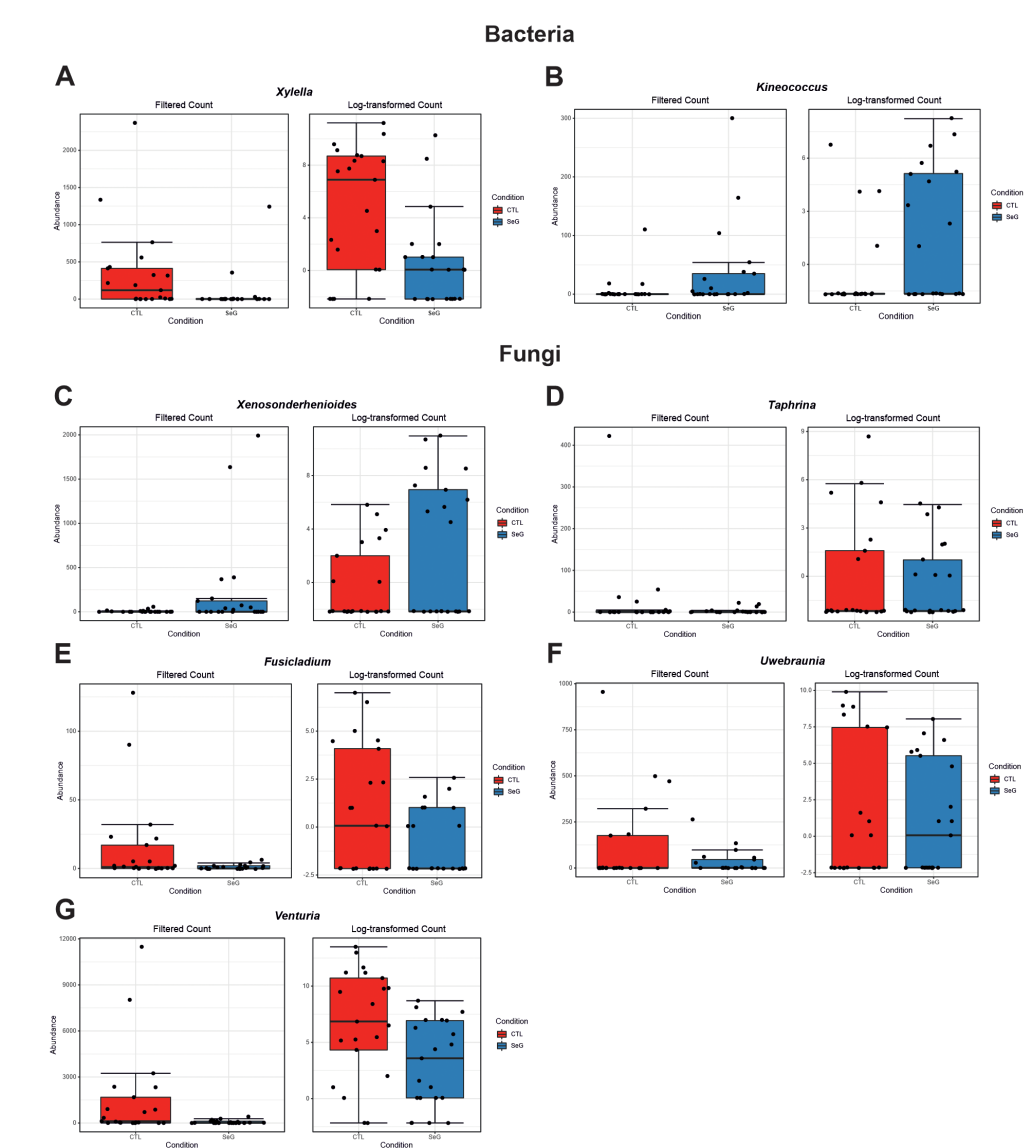
Graphs related to the differentially abundant features between bacteria of CTLs and the SeGs group achieved with the metagenomeSeq method. Genera were ranked according to their FDR value. (**A**, *Xylella*; **B**, *Kineococcus;***C**, *Xenosonderhenioides;***D**, *Taphrina;***E**, *Fusicladium*; **F**, *Uwbraunia;***G**, *Venturia*)

Results from metagenomeSeq analysis allow us to determine and confirm the presence of differentially abundant genera in SeGs and CTLs. Analysis of bacterial data indicated that the CTLs had a significantly higher abundance of the genera *Xylella* (FDR = 0.0167) (Fig. 4A), while *Kineococcus* (FDR = 0.0167) was more abundant in the SeGs (Fig. 4B). By observing fungal data, results indicated that CTLs had a significantly higher abundance of the genera *Taphrina* (FDR = 0.0439), *Fusicladium* (FDR = 0.0439), *Uwebraunia* (FDR = 0.0439), and *Venturia* (FDR = 0.0489) (Fig. 4D-G); while *Xenosonderhenioides* had significantly higher abundance in SeGs (FDR = 0.0002) (Fig. 4C).

**Fig. 5:**
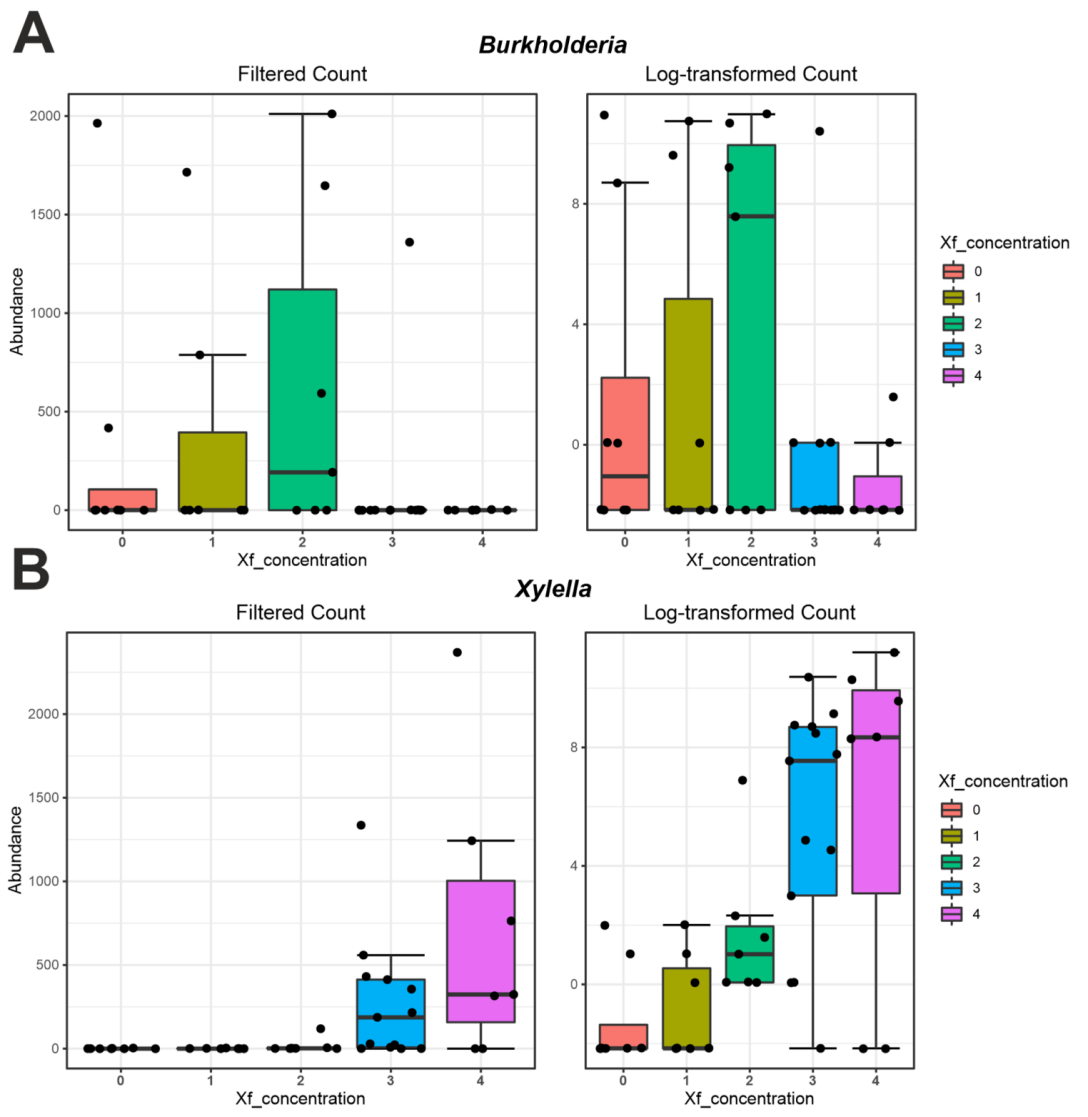
Graph related to the differentially abundant features between bacteria of CTLs and the SeGs group achieved with the DeSeq2 method based on a further variable for sample differentiation represented by the *Xylella fastidiosa* concentration (CFU/mL; 0 = 0-100; 1 = 101 − 10,000; 2 = 10,001-100,000; 3 = 100,001–1,000,000; 4 = 1,000,001-over). Genera were ranked according to their FDR value. (**A**, *Burkholderia*; **B**, *Xylella*)

The identification of differentially regulated OTUs was also assessed using the DeSeq2 method according to a supplementary variable (*Xfp* concentration) for sample differentiation. Data indicates that two genera (*Xylella* and *Burkholderia*) turned into statistically significant and showed an opposite trend based on *Xfp* concentration (Fig. 5). Specifically, *Burkholderia* displayed high number of reads for low *Xfp* concentration (classes 1 and 2) and read detection, whereas displayed low number of reads for high *Xfp* concentration (classes 3 and 4) (Fig. 5), thus indicating a mutualistic exclusion linked to the health plant status.

**Fig. 6:**
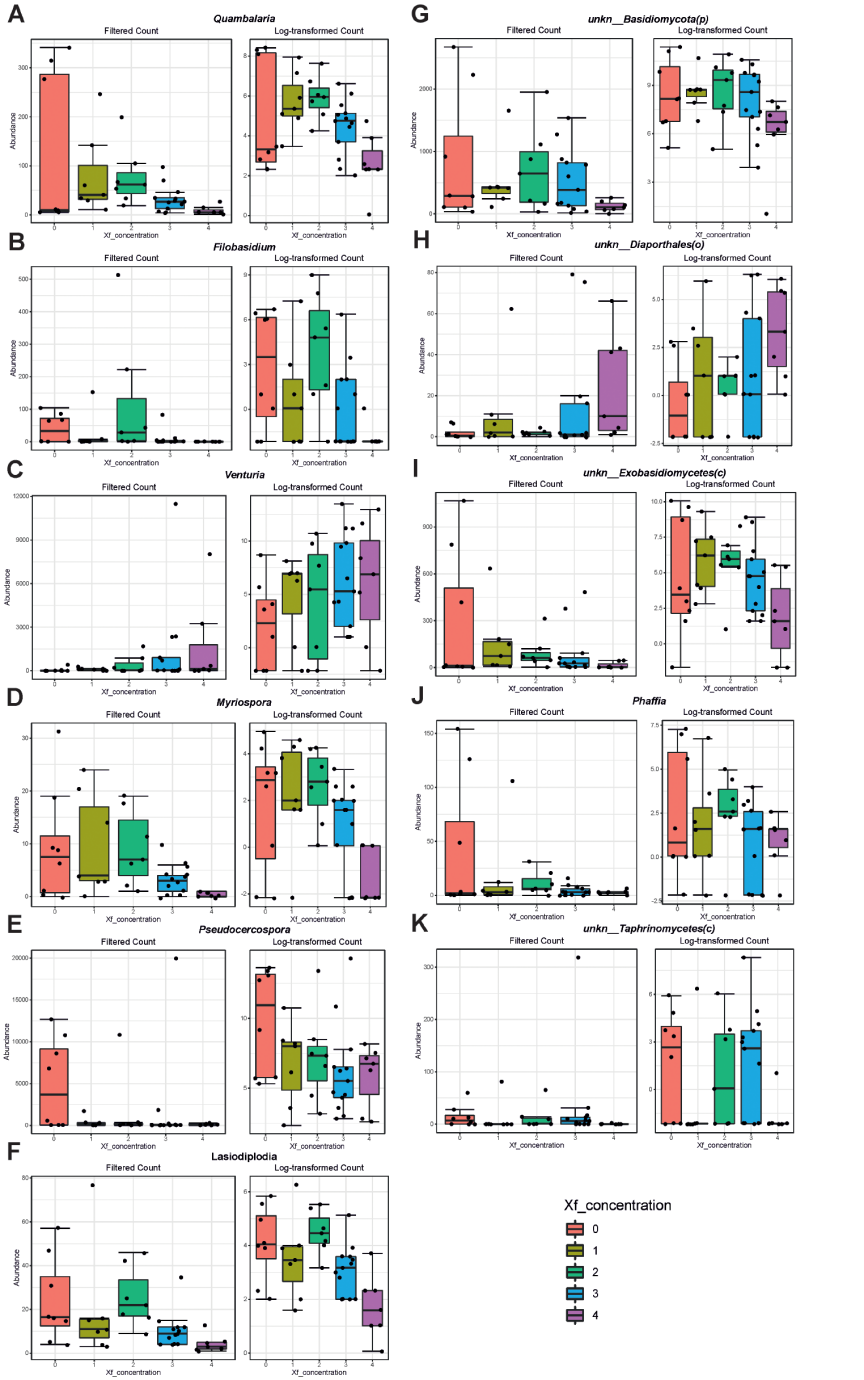
Graph related to the differentially abundant features between fungi of CTLs and the SeGs group achieved with the DeSeq2 method based on a further variable for sample differentiation represented by the *Xylella fastidiosa* concentration (CFU/mL; 0 = 0-100; 1 = 101 − 10,000; 2 = 10,001-100,000; 3 = 100,001–1,000,000; 4 = 1,000,001-over). Genera were ranked according to their FDR value. (**A**, *Quambalaria*; **B**, *Filobasidium*; **C**, *Venturia;***D**, *Myriospora*; **E**, *Pseudocercospora*; **F**, *Lasiodiaplodia*; **G**, unkn. *Basidiomycota;***H**, unkn. *Diaporthales;***I**, unkn. *Exobasidiomycetes*; **J**, *Phaffia*; **K**, unkn. *Taphrinomycetes*)

As for results obtained with fungal data, nine taxa (*Quambalaria*, *Filobasidium*, *Myriospora*, *Pseudocercospora*, *Lasiodiaplodia*, *Phaffia* and undetermined ones within *Basidiomycota*, *Exobasidiomycetes*, *Taphrinomycetes*) and two taxa (*Venturia* and undetermined one within *Diaporthales*) were linked to lower (Fig. 6A, B, D, E, F, G, I, J, K) and higher *Xfp* concentrations, respectively (Fig. 6C, H).

**Fig. 7:**
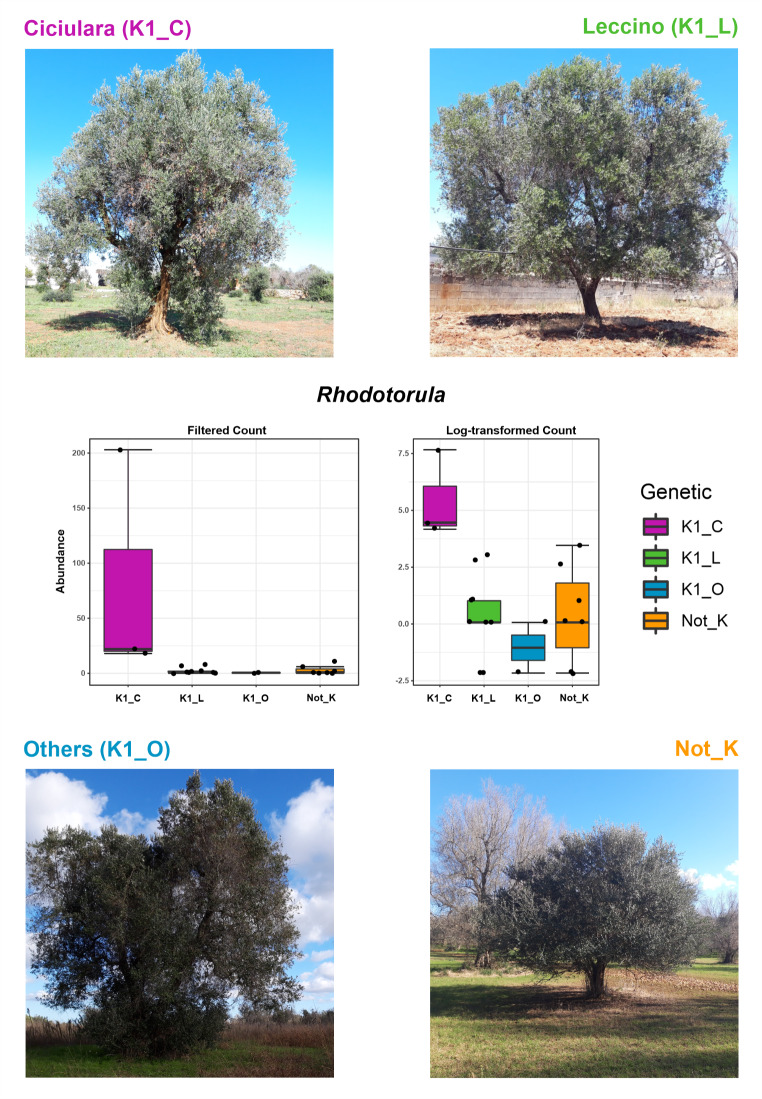
Schematic representation of the *Rhodotorula* genus in SeG samples. Statistical data of the RNAseq method were reported in Table S6. K1_C = genotypes closely related to the cultivar ‘Ciciulara’; K1_L = genotypes closely related with the cultivar ‘Leccino’; K1_O = genotypes grouped in K1 but not attributable to a specific cultivar; Not_K = genotypes not falling in any of the above-mentioned clusters

Data were then further analyzed by selecting only libraries related to SeG samples. The aim of this analysis was to differentiate genera in relation with genetic distance among SeGs, which was previously determined by Pavan et al. (2021) [21]. Results reported in Table S6, according to DESeq2 method, indicate that statistically significant genera were reported for both datasets (Bacteria and Fungi). *Rhodotorula* (Fig. 7) represented the most significant taxon (log_2_FC=-54.356 FDR = 0.019984; Tab. S6B), whose occurrence was only associated with SeG samples belonging to the Ciciulara genetic cluster, previously described by Pavan et al. 2021 [21].

### Co-occurrence networks

**Fig. 8:**
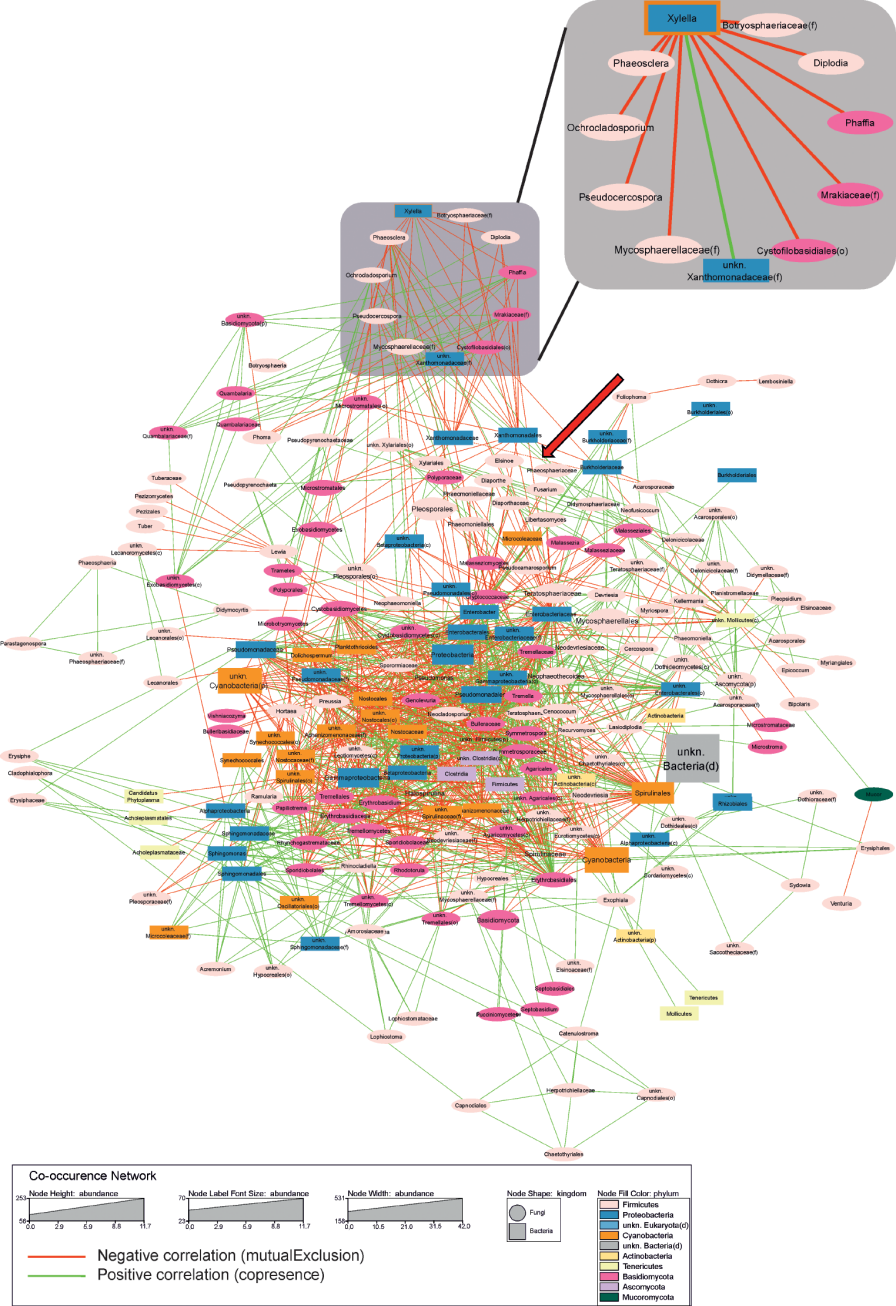
Co-occurrence network of the endophytic microbiota of leaves infected with *Xylella fastidiosa*. Node shape represents kingdom (squares = bacterial taxa, ovals = fungal taxa), while node size, label font size and node width represent the abundance (number of reads) according to the legend. Nodes were colored by phylum and labeled according to the lowest identified taxonomic level. Edges (connections) are colored green (positive) or red (negative). The zoom of *Xf*-cluster is separately in the top right (circular layout with grey background). Red cross highlights the occurrence of a specific negative relationship (*Burkholderiaceae*-*Xanthomonadaceae*).

The bacterial and fungal microbiome were also tested to identify all the potential interactions according to a co-occurrence network. Using the Cytoscape software coupled with the CoNet package, out of 66 bacterial and 165 fungal OTUs passes the minimum threshold showing at least one potential interactor in the network. These interactions were supported by at least three out of four computation methods used. In detail, 1380 potential interactions (also called edges) were computed, 641 of which are negative (mutual exclusion) and 739 positives (copresence). Furthermore, the description of the relationships between *Xfp* and the potential interactors was improved by a specific focus reported in Fig. 8 (upper-right). This focus allows a straightforward interpretation of network results, thus indicating as *Xfp* directly interacts with ten genera. Among them, the only positive bacterial interaction is with an unidentified bacterium belonging to the Xanthomonadaceae family. Furthermore, the pattern showed that nine fungal genera are negatively correlated with *Xylella*, indicating an inter-kingdom interaction: *Phaeosclera*, *Phaffia*, *Mrakiaceae*, *Cystofilobasidiales*, *Pseudocercospora*, *Ochrocladodporium*, *Botryosphaeriaceae*, *Diploidia*, *Mycosphaerellaceae* (Fig. 8).

## Discussion

The current lack of an effective method for pathogen management, the increasing demand for ready-to-use solutions to restore the territory landscape and the olive-growing economic sector of Apulia have pushed research towards the identification of germplasm displaying tolerant or resistant phenotype to *Xfp* infection. A few years after the start of the epidemic, it was possible to observe the presence of asymptomatic or low symptomatic genotypes in highly compromised olive groves, including the Leccino and FS17 cultivars that represent cases of resistance [22]. Several defence mechanisms, including biochemical responses and plant structural characteristics, have been proposed to contribute to the resistance of different genotypes against *Xfp*. Those mechanisms may operate within the xylem tissues contributing to reduce systemic colonization by the pathogen and may include: the build-up of vessel occlusions by gums or tyloses, the accumulation of phenolics and lignin, the induction of pathogenesis-related proteins, antioxidant-related enzymes and the variation in the ionome content [23–26]. A few studies described the microbiome community and the activity of some bacterial isolates against *Xfp* [16, 17], still there is a lack of information on the endophytic communities in asymptomatic or low symptomatic olive genotypes occurring in heavily infected orchards of Salento area which may have originated from sexual reproduction or represent clonal variants of known cultivars [21].

This gap was bridged by the present work, where the information about the involvement of endophytic communities in counteracting the *Xfp* related disease was deepened and then associated with genetic resources collected during exploratory missions in olive orchards severely affected by the OQDS. Cyanobacteria represent the most abundant phylum identified in the present work. Metagenomic analyses have shown a diversity of cyanobacterial phylotypes as common endophytes of high-valued plant species like rice, wheatgrass, and Jingbai pear trees [27, 28]. This group of bacteria is able to establish nitrogen-fixing symbiotic associations with representatives of all four major phylogenetic divisions of terrestrial plants [29]. These results could underline that the presence of Cyanobacteria inside plants is increased under nutrient-limiting conditions [30], such as those caused by *Xfp* infection.

Our results suggest that the occurrence of *Xfp* reshapes the composition of endophytes by altering microbial structure and diversity. In this view, some bacterial and fungal taxa appear to be statistically significant in the CTL and SeG comparison, whose presence was also confirmed in our previous study [25] despite not having been described in-depth, as these taxa did not appear as statistically significant in the previous experimental design (21 vs. 1 SeG samples). Indeed, CTL plants showed a dramatic reduction of the bacterial genera predominant in SeG vessels (i.e., *Burkholderia*, *Dolichospermun*, *Bacillus, Hymenobacter*, and *Sphingomonas*). The same bacterial genera were previously reported in other plant species, including potato [31], sugarcane [32], or citrus [33], apple [34], and maple trees [35], and more specifically as common xylem-inhabitants of woody crops including olive and grapevine [36]. Bacterial species belonging to the *Burkholderia* genus are ubiquitous in soil, mostly in the rhizosphere [37], and were reported to heavily colonize at least 30 plant species, as well as the fruiting bodies of *Tuber magnatum* [38]. Here, we found a positive association between the genus *Burkholderia* and the SeGs group, which was confirmed by the results from DeSeq2 analysis using *Xfp* concentration as a supplementary variable for computation (Fig. 5). In addition, we found a mutualistic exclusion between *Burkholderia* and *Xylella*. *Burkholderia* reaches the highest level in stage 3 (CFU/mL = 100,001–1,000,000), thus highlighting as it could contrast the presence of the pathogen until a threshold limit. While this effect may not seem to be confirmed by the network analysis, indicating that *Burkholderia* does not have a direct link to *Xylella* (Fig. 8), a more thorough investigation of the network suggests an indirect action for mitigating the pathogen spread [39]. Indeed, *Burkholderia* shows a direct (negative) correlation with Xanthomonadaceae family, which has, in turn, a direct (positive) correlation with *Xylella*. The role of *Burkholderi*a spp. against plant pathogens is currently well-known and documented [40], including its role in a more complex PGPR (Plant growth-promoting rhizobacteria) network for better exerting its anti-pathogen activity. Overall, we suggest that *Burkholderia* spp., that can develop and compete for nutrients and colonization in the xylem vessels, could enact survival strategies that can help the plant health, such as the production of antibiotics, enzymes, and bacteriolytic molecules, similarly to those they deploy when colonizing the rhizosphere [41]. The other taxa positively associated with the SeGs group are *Dolichospermum* and *Bacillus* spp., previously inserted in the list of bacteria producing siderophores, salicylic acid, cytokinins, and able to phosphate solubilization in the treatment against *Botrytis cinerea* and *Pseudomonas syringae* [42]. Also, pathogenic bacteria such as *Ralstonia solanacearum*, *Pseudomonas savastanoi* and *Xanthomonas axonopodis* infect plants and generate diseases, whereas *Bacillus* spp. inoculation suppresses pathogen growth and protects plants from diseases [43]. *Bacillus*-induced stress mitigation in crop plants biosynthesis of polyphenol compounds (lignin, flavonoids, and phenylpropanoids) and triggering the plant resistance against environmental stimuli [44]. *Sphingomonas* and *Hymenobacter* have been described as bacteria with several functional plant beneficial traits, including plant growth promotion and plant protection and bioremediation of heavy metal pollution in natural soils [45, 46]. Conversely, when observing results from metagenomeSeq analyses (Fig. 4), only two differently significant bacteria genera were observed *Kineococcus* and *Xylella*. The presence of *Kineococcocus* in the SeGs group would seem to be mirroring the presence of the pathogen *Xylella* in the CTLs group. Cultivated members of *Kineococcus* genera had been previously isolated from plant stems [47] and were identified as members of the Actinobacteria phylum; they have been proposed to increase agricultural productivity through plant-growth promotion and to have the potential to be used as an alternative to chemical fertilizers [48].

Changes were also observed in xylem-associated fungal communities, as genera like *Lembosiella*, *Myriospora*, *Recurvomyces*, *Quambalaria*, *Phaffia*, and *Phaephleospora* were positively associated with SeG group. Based on our data, the fungal endophytic community would seem to have a dominant role with respect to the bacterial one, both in terms of quantity (number of reads) and quality (number of identified genera); this trend also reflects a fundamental antagonism to the presence of the pathogen *Xylella*. In this view, the presence of *Quambalaria* and *Phaffia* is relevant in SeGs and their significance was also confirmed by DeSeq2 analyses using the additional variable for sample differentiation (*Xfp* concentration, Fig. 6). Furthermore, the use of endophytic fungi against olive pathogenic fungi like *Colletotrichum acutatum*, with the aim to control its growth, sporulation, germination, or cause hyphae abnormalities in vitro conditions, has also been recently reviewed [49]. Some of these fungal endophytes include *Alternaria* sp., *Diaporthe* sp., *Nigrospora oryzae*, *Chondrostereum purpureum*, *Chaetomium globosum*, *Quambalaria cyanescens*, *Epicoccum nigrum*, and several *Aspergillus* species. The inhibitory effect explicate by those endophytes was ascribed to the production and release of various volatile organic compounds, such as phenylethyl alcohol, benzothiazole, 4-methylquinazoline, lilial, galaxolide and benzyl alcohol [50].

Two of the main findings of the present work are the identification, in SeGs, of the genus *Rhodotorula* and *Phaffia*.

*Rhodotorula* is a fungal genus that was only identified in SeG plants, mostly in the Ciciulara group samples. Species belonging to this genus show different lifestyles, which can change basing on the host species. In humans, they are considered opportunistic pathogens whereas in plants they can have negative or beneficial effects. In the latter case, some consistent examples are represented by *Rhodotorula graminis* strain WP1 v1.1, a yeast involved in the auxin production in poplar species [51], *R. mucilaginosa*, which exerts a biocontrol activity against fungal and bacterial species in rice [52] and *R. dairenensi* isolated in CVC-asymptomatic sweet orange plants and tangerine (*C. reticulata*) plants [53]. *Rhodotorula* was also already identified in olive tree microbiota, mostly in the cultivar Cobrançosa [54]. Despite that, their lifestyle strategies and how these species interact with plants is a topic not been deeply investigated [53], still representing an interesting research field for future applications. The second finding, the identification of the genus *Phaffia*, was also confirmed by the results of a robust co-occurrence analysis (Fig. 7) led to highlighting a negative correlation between *Phaffia* and *Xfp.*

The *Phaffia* genus consists of only one *Phaffia rhodozyma* species, which is involved in astaxanthin production [55]. The antimicrobial activity of astaxanthin was previously associated with the protection of human gastric epithelial cells from the harmful effects of *Helicobacter pylori* infection, with a mechanism based on the attenuation of reactive oxygen species (ROS) production and the increase of antioxidant enzyme activity [55]. Thus, astaxanthin may contrast the action of oxidative enzymes, which are known to generate ROS-mediated cell wall degradation by plant pathogens [56]. Novelli et al. (2019) [57] reported that Leccino samples have a higher concentration of ROS compared to Cellina di Nardò, an olive cultivar susceptible to *Xf*. In this way, the increased production of ROS may not just switch on plant defence signaling pathways; it may represent a key factor for increasing the astaxanthin production *P. rhodozyma* in counteract *Xf* infection. The role of ROS as a trigger for the production of antimicrobial-based compounds was previously reported by Chauhan et al. (2022) [58] where the presence of environmental factors like high light radiation causes elevated levels of ROS, which in turn promotes the accumulation of astaxanthin [59]. Overall, our results suggest an antagonist role by the fungal endophytic community, previously associated with the protection of plants from abiotic and biotic stresses, and enhanced plant competitive ability [60]. In addition, they provide a basis for unraveling the impact of *Xfp* infection on the xylem microbial communities, and for the identification of microorganisms associated with tolerant or resistant genotypes. As previously shown for the *Paraburkholderia phytofirmans* strain PsJN, which appears highly efficient for the control or eradication of the Pierce’s disease [61], the genera *Burkholderia*, *Quambalaria*, *Phaffia* and *Rhotodorula* might be of interest to the development of defence strategies against *Xfp*. Nevertheless, although the identification of most of these species was also reported in our previous work [25], further analyses are ongoing to evaluate the effect of these putatively beneficial species in contrasting the *Xfp* infection.

## Conclusions

Endophytic microbiota mediates the interactions between the plant and other microorganisms through competition for nutrients and spaces, synthesizing molecules with antibiotic action or activating the plant’s systemic defences. In our work, the relationships between bacterial and fungal species were first analyzed separately and then through correlation analysis to resolve the intricate relations system between the identified OTUs of both kingdoms.

Our work highlighted the presence of a specific community associated with the SeGs, thus indicating the presence of well-adapted genetic resources able to survive after years of pathogen-applied pressure, according to the detection of microorganisms of the genera *Burkholderia*, *Quambalaria*, and *Phaffia*. Also, the identification of *Rhodotorula* represents an interesting starting point for developing synthetic community to improve the natural resistance of olive tree. The pioneering proposal of this work on the olive-*Xf* pathosystem demonstrated that in susceptible plants, there is a significant change in the associated microbiota with a drastic loss of beneficial genera. Despite the presented evidence that suggests a role of the endophytic community in this resistance phenomenon, we may not exclude the possibility that the absence of helpful microorganisms in CTL plants may be caused by the presence of *Xfp* itself, being an effect rather than a cause of the pathogen’s spread.

This is the first study that provides an overview of endophytic communities associated with several putatively resistant olive genotypes located in areas under high *Xf* inoculum pressure. While aware that approaches such as artificial inoculations in controlled environments can guarantee much more reliable results, we are confident that the data presented in this study will be useful to the scientific community, as well as the whole agricultural sector. Identifying these negatively correlated genera provides valuable insights into the potential antagonistic microbial resources and their possible development as biocontrol agents against *Xf*, which could contribute to the development of novel strategies for disease management. Future studies could maximize their beneficial effects to help farmers select the more appropriate genetic resource to restore the olive growing and the area’s landscape.

## Methods

### Plant material

Twenty-one selected genotypes (SeGs), previously described as asymptomatic or low symptomatic in olive groves heavily compromised by *Xfp* [21], were used as starting material of this study. SeGs displayed a disease severity index < 1.5, based on the 0–3 using a rating scale proposed by Luvisi et al. (2017) [23]. From each SeG, about one-year-old twigs with attached leaves were collected from several canopy points (apical, median, and basal portion), placed in plastic bags, and labelled for their registration and traceability once in the laboratory. In addition, 63 olive trees (disease severity index ≥ 1.5) belonging to susceptible cultivar Cellina di Nardò were also collected. For each SeG has been defined a CTL obtained from a mix of three infected plants randomly chosen in the proximity of each SeG to represent the microbiome in susceptible cultivars in the same area. *Xf* concentration (C), expressed as bacterial cfu ml^− 1^, was inferred by the standard calibration curve, using Cqs from qPCR as described by D’Attoma et al. (2019) [36]. Information describing SeG properties in this study is obtained from Pavan et al. (2021) [32] and is reported in Tables S7 and S8.

### DNA extraction, amplification, and endophyte sequencing

Before DNA extraction, samples were washed with deionized water, surface-sterilized by sequential immersion in ethanol 70% (v/v) for 1 min, bleach (3–5% chlorine) for 5 min, and ethanol 70% (v/v) for 30 s, and washed three times with sterile deionized water. Approximately 1 g of leaf petioles from each SeG sample were transferred into an extraction bag (BIOREBA, Switzerland) and subjected to DNA extraction according to the protocol of Edwards and co-workers (1991) [27]. The same procedure was applied to CTL samples obtained by pooling petioles from three olive trees of the Cellina di Nardò cultivar located in proximity to the corresponding SeG sample.

The extracted DNA was used to perform a two-step amplification protocol in which the core PCR primer and the adaptors CS1 (forward) and CS2 (reverse) were included in a single oligonucleotide as previously described by Vergine et al. (2020) [16]. The oligonucleotide sequences were (core PCR primer in bold) 5.8S-Fun (5-ACACTGACGACATGGTTCTACA-**AACTTTYRRCAAYGGATCWCT**-3’) and ITS4-Fun (5’-TACGGTAGCAGAGACTTGGTCT-**CCTCCGCTTATTGATATGCTTAART**-3’) [62] for fungi, 341 F (5’ ACACTGACGACATGGTTCTACA-**CCTAYGGGDBGCWSCAG**-3’) and 806R (5’-TACGGTAGCAGAGACTTGGTCT-**GGACTACNVGGGTHTCTAAT**-3’) for prokaryotes [63]. A mixture of peptide nucleotide acid (PNA) blockers oligos (PNA Bio Inc., Thousand Oaks, CA,

USA) targeted at plant mitochondrial and plastidic genomes was added, to increase the fraction of.

bacterial sequences, reduce the PCR-bias, and thus result in more accurate sequencing [63, 64]. The PCR assay was carried out in triplicate using DNA samples extracted following the protocols described by Vergine et al. (2020) [16], and the integrity and quality of the PCR products were checked on an agarose gel. PCR replicates were pooled and sequenced on an Illumina MiSeq platform (v3 chemistry) at the Génome Québec Innovation Center (McGill University, Montréal, Canada).

Metabarcoding libraries are available at 10.6084/m9.figshare.20448513 (bacteria) and 10.6084/m9.figshare.20448540 (fungi).

### Bioinformatic analysis of the sequences

Raw reads were processed with FASTQC (https://www.bioinformatics.babraham.ac.uk/projects/fastqc/) to evaluate sequencing quality. Then the software Trimmomatic (http://www.usadellab.org/cms/page=trimmomatic) [65] was used to remove adapters and low-quality bases using a windows-based approach, by setting a window size of 5 bp and a minimum quality of 20. In addition, reads shorter than 35 bp were removed. Finally, trimmed data were processed with the Metagenomics GAIA 2.0 tool (http://www.metagenomics.cloud, Sequentia Biotech, Barcelona, Spain, 2017) using a database of all the classified 16 S or ITS sequences downloaded from the NCBI nr database on February 2021. The identities used to classify the reads into the different taxonomic categories (domain, phylum, class, order, family, genus, species) were 75, 78, 85, 89, 91, 93, and 97%, respectively. Uniquely mapped reads classified in a species are also classified in the strain of the reference they map.

Bacterial and fungal community profiling was also performed using the GAIA pipeline by α- and β-diversity analyses. In particular, the diversity within the samples (*α*-diversity) was calculated using the Chao1, Shannon, Fischer, and Simpson indices. The diversity among samples (*β*-diversity) was calculated by Bray Curtis index and visualized in a two-dimensional principal component analysis (PCA). An adjusted p-value cutoff (FDR) of 0.05 was considered in both cases.

The output from GAIA (OTU table and related taxonomy) was converted to a .biom file and used with MicrobiomeAnalyst [66] for data visualization and statistical assessment. Data were filtered to remove low-quality and not informative features as described by Sillo et al. (2022) [38].

The differential abundance analysis analyses were carried out by DESeq2 method (FDR = 0.05) and performed by grouping samples according to two additional variables. The first one was based on the *Xfp* concentration data (Table S7) obtained through qPCR, whose range were manually determined (CFU/mL; 0 = 1-100; 1 = 101 − 10,000; 2 = 10,001-100,000; 3 = 100,001–1,000,000; 4 = 1,000,001-over), and the latter that describes the genetic group associated with each SeG, whose information is provided by Pavan et al. (2021) [21] and reported in Table S8. Specifically, according to DNA fingerprinting and hierarchical clustering by Pavan et al. (2021) [21], most of the SeGs were assigned to the K1 genetic cluster. Within K1, three genotypes (SX25, 27, 29) grouped in a subcluster closely related to the local cultivars Ciciulara (K1_Ciciulara) and nine SeGs (SX31, 61, 65, 67, 77, 79, 81, 83, 89) grouped in a sub-cluster of genotypes closely related to the cultivar Leccino (K1_Leccino). The other three plants (SX73, 75, 87) assigned to the K1 genetic cluster could not be related to Leccino or Ciciulara but to other cultivars (K1_Others). The six remaining SeGs (SX30, 32, 71, 69, 63, 85) were not clusterized into any group (Not_K).

### Co-occurrence network

Potential bacterial and fungal interactors with *Xfp* were identified using the software packages Cytoscape (version 3.9.1) [67] and CoNet package (version 1.1.1 beta) [68]. Non-rarefied OTU tables were used as input tables and then coupled, then prefiltered based on the sum of each OTUs that should be equal to or larger than a specified value (row_miniocc = 30). Data were then standardized using the column normalization option (col_norm), where each column of data is divided by its sum, then abundances were converted using the column-wise proportions. Statistically significant OTUs were then assessed using four different p-value basing methods: Pearson and Spearman (correlations based) and Bray Curtis and Kullback-Leibler (dissimilarities based). The following threshold settings were applied: edge selection based on edge number limiting the edge number to 2000 using the top and bottom. This data was selected according to preliminary matrix analyses (Compute matrix info). The Force intersection option was enabled.

Data were merged based on the mean, using the min.support option (minimum number of methods for supporting edges = 3). Next, 100 iterations were calculated within each method using the edgescores routine, with bootstrap option for resampling. Data from p-value basing methods were then merged using the p-value option (brown method) by filtering the uneven edges. Finally, the Benjamini Hochberg test was used, and multiple test correction for OTU selection was achieved by setting 0.05 as the threshold.

### Electronic supplementary material

Below is the link to the electronic supplementary material.

Supplementary Material 1

Supplementary Material 2

Supplementary Material 3

Supplementary Material 4

Supplementary Material 5

Supplementary Material 6

Supplementary Material 7

Supplementary Material 8

Supplementary Material 9

## Data Availability

The datasets presented in this study can be found in online repositories. The names of the repository/repositories and accession number(s) can be found below: https://figshare.com/; 10.6084/m9.figshare.20448513 (bacteria); 10.6084/m9.figshare.20448540 (fungi).

## References

[1] Singh R, Dubey AK (2015). Endophytic actinomycetes as emerging source for therapeutic compounds. Indo Glob J Pharm Sci.

[2] Hu T, Chitnis N, Monos D, Dinh A (2021). Next-generation sequencing technologies: an overview. Hum Immunol.

[3] Nair DN, Padmavathy S. Impact of endophytic microorganisms on plants, environment and humans. Sci World J. 2014;2014. 10.1155/2014/250693 PMC3920680 24587715

[4] Upadhyay SK, Rajput VD, Kumari A, Espinosa-Saiz D, Menendez E, Minkina T (2022). Plant growth-promoting rhizobacteria: a potential bio-asset for restoration of degraded soil and crop productivity with sustainable emerging techniques. Environ Geochem Health.

[5] Vita F, Sabbatini L, Sillo F, Ghignone S, Vergine M, Nissim WG (2022). Salt stress in olive tree shapes resident endophytic microbiota. Front Plant Sci.

[6] Upadhyay SK, Srivastava AK, Rajput VD, Chauhan PK, Bhojiya AA, Jain D et al. Root exudates: mechanistic insight of Plant Growth promoting Rhizobacteria for sustainable crop production. Front Microbiol. 2022;13 July. 10.3389/fmicb.2022.916488 PMC9329127 35910633

[7] Jahn L, Hofmann U, Ludwig-Müller J. Indole-3-acetic acid is synthesized by the endophyte C*yanodermella asteris* via a tryptophan-dependent and-independent way and mediates the interaction with a non-host plant. Int J Mol Sci. 2021;22:1–19. 10.3390/ijms22052651 PMC7961953 33800748

[8] Saponari M, Giampetruzzi A, Loconsole G, Boscia D, Saldarelli P (2019). *Xylella fastidiosa* in olive in apulia: where we stand. Phytopathology.

[9] Sabella E, Pierro R, Panattoni A, Materazzi A, Vergine M, De Bellis L (2018). Physiological and molecular Plant Pathology E Ff ects of modulation of potassium channels in tobacco mosaic virus elimination. Physiol Mol Plant Pathol.

[10] Lindow S (2019). Money matters: fueling rapid recent insight into *Xylella fastidiosa* — an important and expanding global pathogen. Phytopathology.

[11] Semeraro T, Gatto E, Buccolieri R, Catanzaro V, De Bellis L, Cotrozzi L et al. How Ecosystem Services can strengthen the regeneration policies for monumental Olive Groves destroyed by *Xylella fastidiosa* Bacterium in a Peri-urban Area. 2021;:1–22.

[12] Quetglas B, Olmo D, Nieto A, Borràs D, Adrover F, Pedrosa A et al. Evaluation of Control Strategies for *Xylella fastidiosa* in the Balearic Islands. Microorganisms. 2022;10. 10.3390/microorganisms10122393 PMC9780951 36557646

[13] Cabezas-Cruz A, Maitre A. Is Plant Microbiota a driver of resistance to the Vector-Borne Pathogen *Xylella fastidiosa*? Pathogens. 2022;11:1–4. 10.3390/pathogens11121492 PMC9782604 36558826

[14] Vayssier-Taussat M, Albina E, Citti C, Cosson JF, Jacques MA, Lebrun MH (2014). Shifting the paradigm from pathogens to pathobiome new concepts in the light of meta-omics. Front Cell Infect Microbiol.

[15] Anguita-Maeso M, Trapero-Casas JL, Olivares-García C, Ruano-Rosa D, Palomo-Ríos E, Jiménez-Díaz RM, et al. *Verticillium dahliae* inoculation and in vitro propagation modify the xylem microbiome and disease reaction to Verticillium Wilt in a wild olive genotype. Front Plant Sci. 2021;12:1–15. 10.3389/fpls.2021.632689 PMC7966730 33747012

[16] Vergine M, Meyer JB, Cardinale M, Sabella E, Hartmann M, Cherubini P et al. The *Xylella fastidiosa*-resistant olive cultivar leccino has stable endophytic microbiota during the olive quick decline syndrome (OQDS). Pathogens. 2020;9. 10.3390/pathogens9010035 PMC7168594 31906093

[17] Anguita-Maeso M, Ares-Yebra A, Haro C, Román-Écija M, Olivares-García C, Costa J (2022). *Xylella fastidiosa* Infection Reshapes Microbial Composition and Network Associations in the Xylem of Almond Trees. Front Microbiol.

[18] Ru W, Pang Y, Gan Y, Liu Q, Bao J. Phenolic compounds and antioxidant activities of potato cultivars with white, yellow, red and purple flesh. Antioxidants. 2019;8. 10.3390/antiox8100419 PMC6827044 31547004

[19] Hanani A, Valentini F, Sanzani SM, Santoro F, Minutillo SA, Gallo M et al. Community analysis of culturable sapwood endophytes from apulian olive varieties with different susceptibility to *Xylella fastidiosa*. Agronomy. 2022;12.

[20] Mourou M, Hanani A, D’onghia AM, Davino SW, Balestra GM, Valentini F. Antagonism and antimicrobial capacity of epiphytic and endophytic Bacteria against the Phytopathogen *Xylella fastidiosa*. Agronomy. 2022;12.

[21] Pavan S, Vergine M, Nicolì F, Sabella E, Aprile A, Negro C et al. Screening of Olive Biodiversity defines genotypes potentially resistant to *Xylella fastidiosa*. Front Plant Sci. 2021;12 August. 10.3389/fpls.2021.723879 PMC8415753 34484283

[22] Saponari M, Boscia D, Loconsole G, Zicca S, D’Attoma G, Morelli M (2017). Isolation and pathogenicity of *Xylella fastidiosa* associated to the olive quick decline syndrome in southern Italy. Sci Rep.

[23] Luvisi A, Aprile A, Sabella E, Vergine M, Nutricati E, Miceli A (2017). *Xylella fastidiosa* subsp. *pauca* (CoDiRO strain) infection in four olive (*Olea europaea* L.) cultivars: profile of phenolic compounds in leaves and progression of leaf scorch symptoms Phytopathol. Mediterr.

[24] D’attoma G, Morelli M, Saldarelli P, Saponari M, Giampetruzzi A, Boscia D et al. Ionomic differences between susceptible and resistant olive cultivars infected by *Xylella fastidiosa* in the outbreak area of Salento. Italy Pathogens. 2019;8. 10.3390/pathogens8040272 PMC6963573 31795218

[25] De Pascali M, Vergine M, Sabella E, Aprile A, Nutricati E, Nicolì F et al. Molecular effects of *Xylella fastidiosa* and drought combined stress in olive trees. Plants. 2019;8. 10.3390/plants8110437 PMC6918294 31652681

[26] Vergine M, Pavan S, Negro C, Nicolì F, Greco D, Sabella E (2022). Phenolic characterization of olive genotypes potentially resistant to *Xylella*. J Plant Interact.

[27] Edwards J, Johnson C, Santos-Medellín C, Lurie E, Podishetty NK, Bhatnagar S (2015). Structure, variation, and assembly of the root-associated microbiomes of rice. Proc Natl Acad Sci U S A.

[28] Collavino MM, Cabrera EVR, Bruno C, Aguilar OM (2020). Effect of soil chemical fertilization on the diversity and composition of the tomato endophytic diazotrophic community at different stages of growth. Brazilian J Microbiol.

[29] Alvarez C, Navarro JA, Molina-Heredia FP, Mariscal V (2020). Endophytic colonization of rice (*Oryza sativa* L.) by the symbiotic strain nostoc punctiforme PCC 73102. Mol Plant-Microbe Interact.

[30] Kollmen J, Strieth D (2022). The Beneficial effects of Cyanobacterial Co-culture on Plant Growth. Life.

[31] Tangapo AM, Astuti DI, Aditiawati P (2018). Dynamics and diversity of cultivable rhizospheric and endophytic bacteria during the growth stages of cilembu sweet potato (*Ipomoea batatas* L. var. *Cilembu*). Agric Nat Resour.

[32] Singh RK, Singh P, Sharma A, Guo DJ, Upadhyay SK, Song QQ et al. Unraveling Nitrogen fixing potential of endophytic diazotrophs of different Saccharum species for sustainable sugarcane growth. Int J Mol Sci. 2022;23. 10.3390/ijms23116242 PMC9181200 35682919

[33] Ginnan NA, Dang T, Bodaghi S, Ruegger PM, McCollum G, England G (2020). Disease-induced microbial shifts in citrus indicate microbiome-derived responses to huanglongbing across the disease severity spectrum. Phytobiomes J.

[34] Tamošiūnė I, Stanienė G, Haimi P, Stanys V, Rugienius R, Baniulis D (2018). Endophytic *Bacillus* and *Pseudomonas* spp. modulate apple shoot growth, cellular redox balance, and protein expression under in vitro conditions. Front Plant Sci.

[35] Wemheuer F, Wemheuer B, Daniel R, Vidal S (2019). Deciphering bacterial and fungal endophyte communities in leaves of two maple trees with green islands. Sci Rep.

[36] Bruez E, Vallance J, Gautier A, Laval V, Compant S, Maurer W (2020). Major changes in grapevine wood microbiota are associated with the onset of Esca, a devastating trunk disease. Environ Microbiol.

[37] Paungfoo-Lonhienne C, Lonhienne TGA, Yeoh YK, Donose BC, Webb RI, Parsons J (2016). Crosstalk between sugarcane and a plant-growth promoting Burkholderia species. Sci Rep.

[38] Sillo F, Vergine M, Luvisi A, Calvo A, Petruzzelli G, Balestrini R (2022). Bacterial communities in the fruiting bodies and background soils of the White Truffle *Tuber magnatum*. Front Microbiol.

[39] Basu A, Prasad P, Das SN, Kalam S, Sayyed RZ, Reddy MS (2021). Plant growth promoting rhizobacteria (pgpr) as green bioinoculants: recent developments, constraints, and prospects. Sustain.

[40] Wang H, Liu R, You MP, Barbetti MJ, Chen Y (2021). Pathogen biocontrol using plant growth-promoting bacteria (PGPR): role of bacterial diversity. Microorganisms.

[41] Luvizotto DM, Marcon J, Andreote FD, Dini-Andreote F, Neves AAC, Araújo WL (2010). Genetic diversity and plant-growth related features of *Burkholderia* spp. from sugarcane roots. World J Microbiol Biotechnol.

[42] Toribio AJ, Jurado MM, Suárez-Estrella F, López MJ, López-González JA, Moreno J. Seed biopriming with cyanobacterial extracts as an eco-friendly strategy to control damping off caused by *Pythium ultimum* in seedbeds. Microbiol Res. 2021;248 April. 10.1016/j.micres.2021.126766 33873139

[43] Krid S, Triki MA, Gargouri A, Rhouma A (2012). Biocontrol of olive knot disease by *Bacillus subtilis* isolated from olive leaves. Ann Microbiol.

[44] Tanaka Y, Matsuoka M, Yamanoto N, Ohashi Y, Kano-Murakami Y, Ozeki Y (1989). Structure and characterization of a cDNA clone for phenylalanine Ammonia-lyase from cut-injured roots of Sweet Potato. Plant Physiol.

[45] Asaf S, Khan AL, Khan MA, Imran QM, Yun BW, Lee IJ (2017). Osmoprotective functions conferred to soybean plants via inoculation with *Sphingomonas* sp. LK11 and exogenous trehalose. Microbiol Res.

[46] Dimitrijević S, Pavlović M, Maksimović S, Ristić M, Filipović V, Antonović D (2018). Plant growth-promoting bacteria elevate the nutritional and functional properties of black cumin and flaxseed fixed oil. J Sci Food Agric.

[47] Qin S, Li J, Chen HH, Zhao GZ, Zhu WY, Jiang CL (2009). Isolation, diversity, and antimicrobial activity of rare actinobacteria from medicinal plants of tropical rain forests in Xishuangbanna China. Appl Environ Microbiol.

[48] Hamedi J, Mohammadipanah F (2015). Biotechnological application and taxonomical distribution of plant growth promoting actinobacteria. J Ind Microbiol Biotechnol.

[49] Talhinhas P, Loureiro A, Oliveira H (2018). Olive anthracnose: a yield- and oil quality-degrading disease caused by several species of Colletotrichum that differ in virulence, host preference and geographical distribution. Mol Plant Pathol.

[50] Landum MC, Félix M, do R, Alho J, Garcia R, Cabrita MJ, Rei F, et al. Antagonistic activity of fungi of *Olea europaea* L. against *Colletotrichum acutatum*. Microbiol Res. 2016;183:100–8. 10.1016/j.micres.2015.12.001. 10.1016/j.micres.2015.12.001 26805623

[51] Firrincieli A, Otillar R, Salamov A, Schmutz J, Khan Z, Redman RS (2015). Genome sequence of the plant growth promoting endophytic yeast *Rhodotorula graminis* WP1. Front Microbiol.

[52] Sen D, Paul K, Saha C, Mukherjee G, Nag M, Ghosh S (2019). A unique life-strategy of an endophytic yeast *Rhodotorula mucilaginosa* JGTA-S1—a comparative genomics viewpoint. DNA Res.

[53] Gai CS, Lacava PT, Maccheroni W, Glienke C, Araújo WL, Miller TA (2009). Diversity of endophytic yeasts from sweet orange and their localization by scanning electron microscopy. J Basic Microbiol.

[54] Costa D, Fernandes T, Martins F, Pereira JA, Tavares RM, Santos PM et al. Illuminating *Olea europaea* L. endophyte fungal community. Microbiol Res. 2021;245 December 2020. 10.1016/j.micres.2020.126693 33482404

[55] Kim SH, Lim JW, Kim H. Astaxanthin inhibits mitochondrial dysfunction and interleukin-8 expression in *Helicobacter pylori*-infected gastric epithelial cells. Nutrients. 2018;10. 10.3390/nu10091320 PMC6164770 30231525

[56] Schroeder WA, Johnson EA (1995). Carotenoids protect *Phaffia rhodozyma* against singlet oxygen damage. J Ind Microbiol.

[57] Novelli S, Gismondi A, Di Marco G, Canuti L, Nanni V, Canini A (2019). Plant defense factors involved in *Olea europaea* resistance against *Xylella fastidiosa* infection. J Plant Res.

[58] Chauhan S, Mahawar S, Jain D, Udpadhay SK, Mohanty SR, Singh A et al. Boosting Sustainable Agriculture by Arbuscular Mycorrhiza under Stress Condition: Mechanism and Future Prospective. Genet Res (Camb). 2022;2022. 10.1155/2022/5275449 PMC9815931 36619307

[59] Basiony M, Ouyang L, Wang D, Yu J, Zhou L, Zhu M (2022). Optimization of microbial cell factories for astaxanthin production: biosynthesis and regulations, engineering strategies and fermentation optimization strategies. Synth Syst Biotechnol.

[60] Clay K, Holah J (1999). Fungal endophyte symbiosis and plant diversity in successional fields. Science.

[61] Baccari C, Antonova E, Lindow S (2019). Biological control of Pierce’s disease of grape by an endophytic bacterium. Phytopathology.

[62] Taylor DL, Walters WA, Lennon NJ, Bochicchio J, Krohn A, Caporaso JG (2016). Accurate estimation of fungal diversity and abundance through improved lineage-specific primers optimized for Illumina amplicon sequencing. Appl Environ Microbiol.

[63] Lundberg DS, Yourstone S, Mieczkowski P, Jones CD, Dangl JL (2013). Practical innovations for high-throughput amplicon sequencing. Nat Methods.

[64] Cregger MA, Veach AM, Yang ZK, Crouch MJ, Vilgalys R, Tuskan GA (2018). The Populus holobiont: dissecting the effects of plant niches and genotype on the microbiome. Microbiome.

[65] Bolger AM, Lohse M, Usadel B (2014). Trimmomatic: a flexible trimmer for Illumina sequence data. Bioinformatics.

[66] Chong J, Liu P, Zhou G, Xia J (2020). Using MicrobiomeAnalyst for comprehensive statistical, functional, and meta-analysis of microbiome data. Nat Protoc.

[67] Shannon P, Markiel A, Ozier O, Baliga S, Wang N, Ramage JT, Amin D, Schwikowski N, Ideker B (1971). Cytoscape: a Software Environment for Integrated models. Genome Res.

[68] Faust K, Raes J. CoNet app: inference of biological association networks using Cytoscape F1000Research. 2016;5:1–15. 10.12688/f1000research.9050.1 PMC5089131 27853510

