# Supplementary material for: Characterization of the olive endophytic community in genotypes displaying a contrasting response to Xylella fastidiosa
Source: BMC Plant Biol. 2024 Apr 25;24:337. doi: 10.1186/s12870-024-04980-2 (PMC11044560; doi:10.1186/s12870-024-04980-2)
Supplement: Supplementary file 1 — Supplementary Material 1 [file 12870_2024_4980_MOESM1_ESM.pdf]

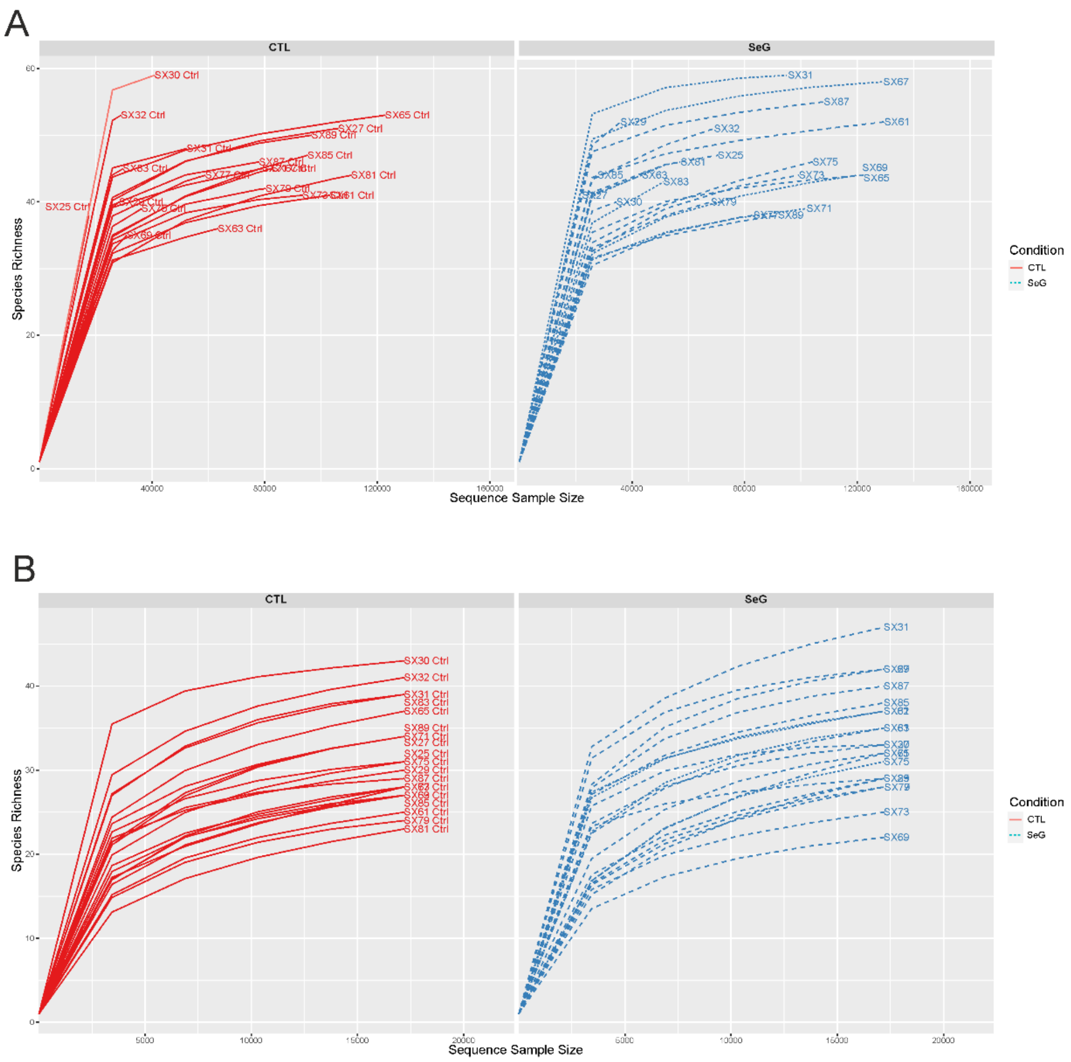

Figure S1. Rarefaction curves of samples grouped according to sample condition before (A, original datasets) and after (B) rarefying to the minimum library size. The horizontal axis indicates the number of reads from Illumina sequencing and the number of sequences obtained through the Illumina MiSeq sequencing platform. The vertical axis shows the number of operational taxonomic units (OTUs) at 99% sequence similarity, approximating the number of identified **bacterial** species.

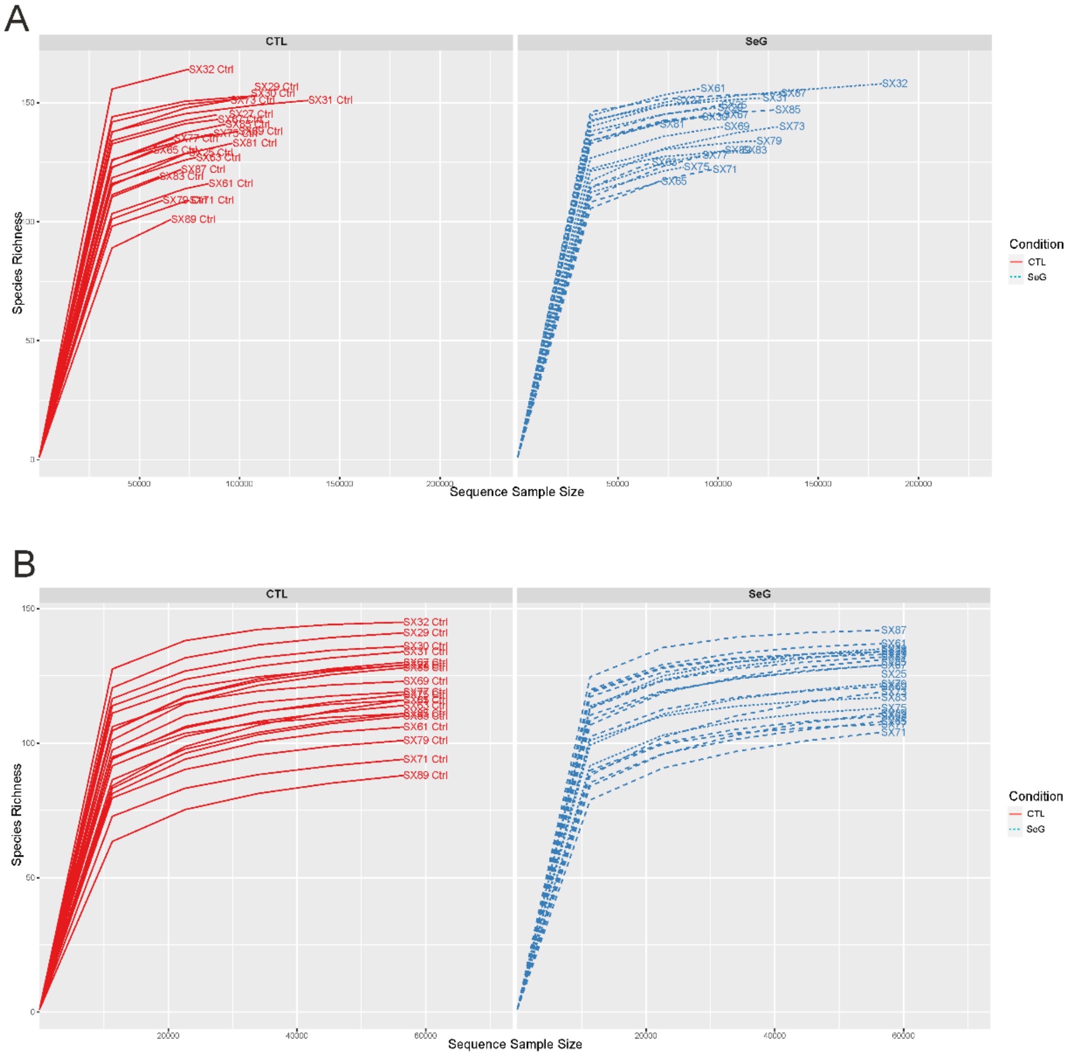

Figure S2. Rarefaction curves of samples grouped according to sample condition before (A, original datasets) and after (B) rarefying to the minimum library size. The horizontal axis indicates the sequences amount getting from Illumina and the number of sequences obtained through the Illumina MiSeq sequencing platform. The vertical axis shows the number of operational taxonomic units (OTUs) at 97% sequence similarity, approximating the number of identified **fungal** species.

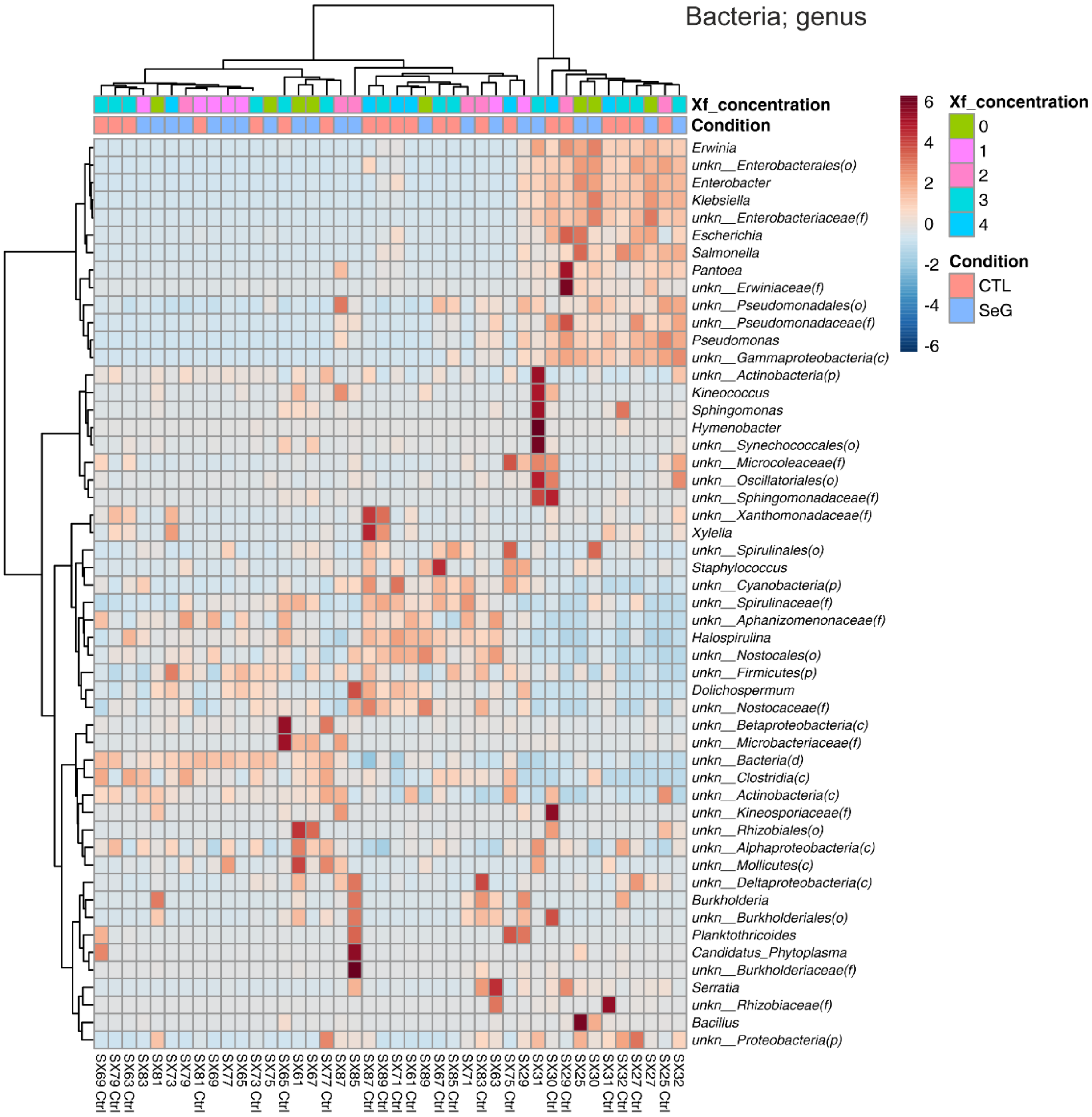

Figure S3. The clustering result is shown as a heatmap using the Euclidean method as distance measure and Ward method as clustering algorithm at the bacterial genus level.

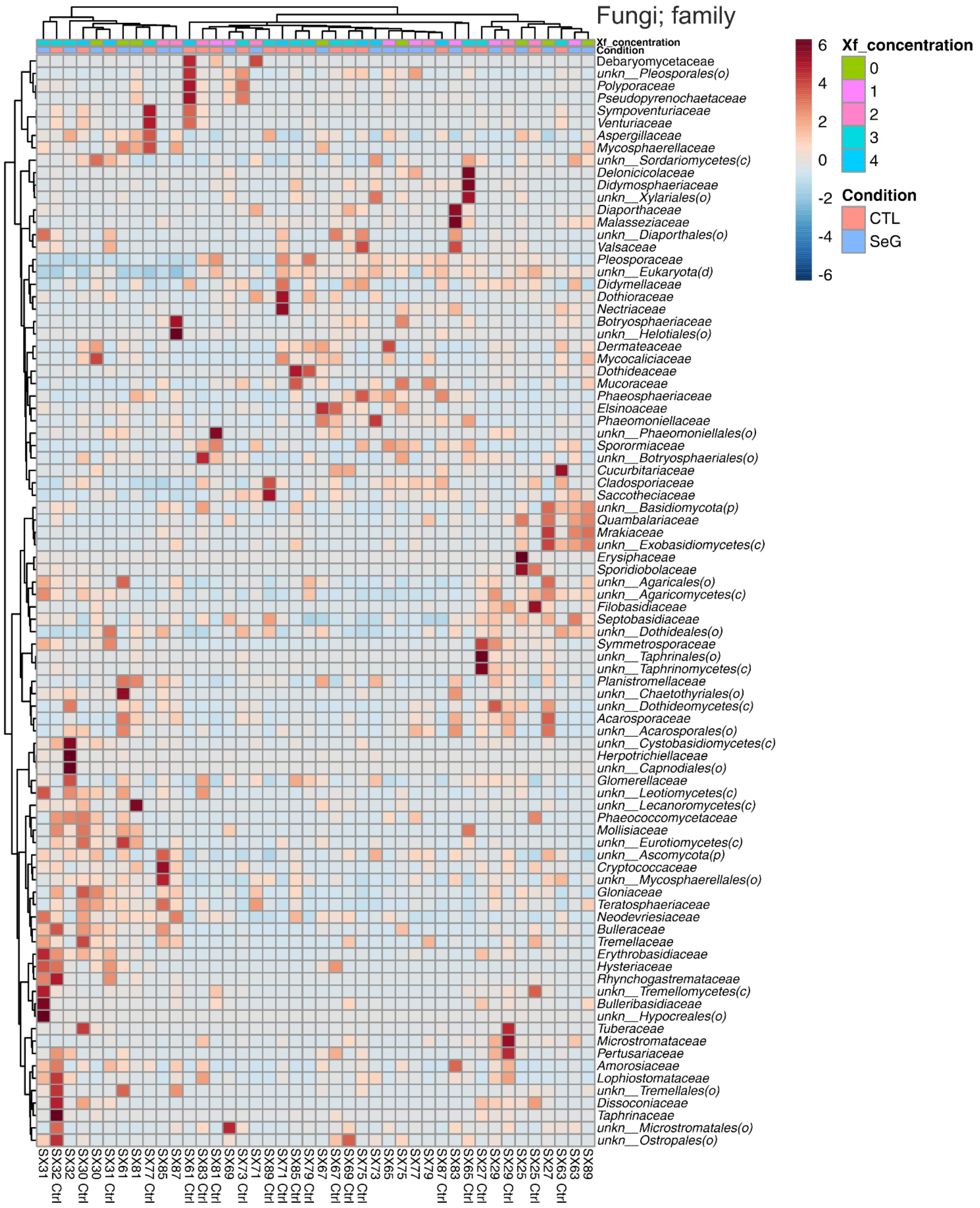

Figure S4. The clustering result is shown as a heatmap using the Euclidean method as distance measure and Ward method as clustering algorithm at the fungal family level.
