# Supplementary material for: Characterization of the olive endophytic community in genotypes displaying a contrasting response to Xylella fastidiosa
Source: BMC Plant Biol. 2024 Apr 25;24:337. doi: 10.1186/s12870-024-04980-2 (PMC11044560; doi:10.1186/s12870-024-04980-2)
Supplement: Supplementary file 4 — Supplementary Material 4 [file 12870_2024_4980_MOESM4_ESM.docx]

**Table S3**. Results of alpha diversity analyses on bacterial (**A**) and fungal (**B**) samples. Data were computed according to four different indexes: Chao1, Shannon, Simpson, and Fisher. SC= Sample code, SeG= Selected genotype; CTL= Control.

| ***A*** | | | | | | | | |
| --- | --- | --- | --- | --- | --- | --- | --- | --- |
| ***Bacteria*** | | | | | | | | |
| **SC** | ***Chao1*** | | ***Shannon*** | | ***Simpson*** | | ***Fisher*** | |
|  | **SeG** | **CTL** | **SeG** | **CTL** | **SeG** | **CTL** | **SeG** | **CTL** |
| ***SX25*** | 48.20 | 41.50 | 1.56 | 1.43 | 0.64 | 0.66 | 4.91 | 4.61 |
| ***SX27*** | 45.00 | 52.25 | 1.49 | 1.65 | 0.68 | 0.72 | 4.86 | 5.13 |
| ***SX29*** | 85.00 | 51.25 | 1.84 | 1.41 | 0.76 | 0.65 | 5.97 | 4.58 |
| ***SX30*** | 41.00 | 60.50 | 1.62 | 1.86 | 0.67 | 0.71 | 4.47 | 6.78 |
| ***SX31*** | 59.33 | 50.00 | 2.15 | 1.67 | 0.79 | 0.69 | 6.11 | 5.21 |
| ***SX32*** | 63.00 | 56.50 | 1.67 | 1.63 | 0.71 | 0.64 | 5.39 | 6.28 |
| ***SX61*** | 57.00 | 43.67 | 1.04 | 1.01 | 0.43 | 0.54 | 5.13 | 4.15 |
| ***SX63*** | 46.50 | 57.00 | 1.50 | 1.01 | 0.68 | 0.54 | 4.83 | 3.69 |
| ***SX65*** | 45.50 | 64.50 | 0.69 | 1.36 | 0.35 | 0.62 | 4.29 | 5.37 |
| ***SX67*** | 58.50 | 60.00 | 0.91 | 1.25 | 0.39 | 0.64 | 5.79 | 4.59 |
| ***SX69*** | 47.50 | 80.50 | 0.57 | 0.59 | 0.28 | 0.26 | 4.30 | 3.90 |
| ***SX71*** | 41.50 | 66.00 | 1.15 | 1.24 | 0.61 | 0.68 | 3.83 | 4.62 |
| ***SX73*** | 47.14 | 41.50 | 0.84 | 0.59 | 0.39 | 0.28 | 4.50 | 4.08 |
| ***SX75*** | 55.00 | 44.60 | 0.60 | 0.98 | 0.30 | 0.50 | 4.59 | 4.32 |
| ***SX77*** | 40.00 | 48.20 | 0.58 | 0.52 | 0.27 | 0.18 | 3.80 | 4.66 |
| ***SX79*** | 49.33 | 43.00 | 0.65 | 0.69 | 0.32 | 0.30 | 4.12 | 4.27 |
| ***SX81*** | 46.86 | 51.20 | 0.99 | 0.51 | 0.45 | 0.24 | 4.91 | 4.34 |
| ***SX83*** | 47.00 | 50.25 | 0.88 | 1.57 | 0.48 | 0.70 | 4.62 | 5.20 |
| ***SX85*** | 46.00 | 53.63 | 1.67 | 1.14 | 0.71 | 0.54 | 5.11 | 4.85 |
| ***SX87*** | 58.33 | 47.20 | 1.14 | 1.59 | 0.50 | 0.76 | 5.57 | 4.74 |
| ***SX89*** | 43.00 | 51.43 | 0.95 | 1.12 | 0.52 | 0.56 | 3.76 | 5.07 |

| ***B*** | | | | | | | | |
| --- | --- | --- | --- | --- | --- | --- | --- | --- |
| ***Fungi*** | | | | | | | | |
| **SC** | ***Chao1*** | | ***Shannon*** | | ***Simpson*** | | ***Fisher*** | |
|  | **SeG** | **CTL** | **SeG** | **CTL** | **SeG** | **CTL** | **SeG** | **CTL** |
| ***SX25*** | 162.20 | 135.00 | 2.39 | 2.00 | 0.75 | 0.67 | 17.15 | 15.17 |
| ***SX27*** | 160.17 | 151.60 | 2.62 | 2.63 | 0.82 | 0.83 | 18.00 | 16.93 |
| ***SX29*** | 151.11 | 155.00 | 2.66 | 2.76 | 0.85 | 0.84 | 17.07 | 17.52 |
| ***SX30*** | 150.00 | 156.20 | 2.56 | 2.94 | 0.83 | 0.89 | 16.72 | 17.50 |
| ***SX31*** | 153.67 | 157.00 | 3.09 | 2.72 | 0.92 | 0.85 | 17.13 | 16.81 |
| ***SX32*** | 176.33 | 169.00 | 2.74 | 3.19 | 0.89 | 0.93 | 17.03 | 19.93 |
| ***SX61*** | 167.00 | 127.38 | 2.98 | 1.76 | 0.90 | 0.70 | 18.33 | 13.24 |
| ***SX63*** | 138.60 | 148.00 | 2.35 | 2.62 | 0.78 | 0.83 | 14.86 | 14.82 |
| ***SX65*** | 130.00 | 136.50 | 2.59 | 2.52 | 0.83 | 0.79 | 13.66 | 15.89 |
| ***SX67*** | 146.15 | 145.80 | 2.59 | 2.67 | 0.83 | 0.83 | 16.61 | 16.66 |
| ***SX69*** | 146.11 | 142.09 | 2.50 | 2.64 | 0.83 | 0.83 | 15.96 | 15.77 |
| ***SX71*** | 131.10 | 126.50 | 2.11 | 1.97 | 0.78 | 0.67 | 13.77 | 12.53 |
| ***SX73*** | 148.27 | 156.00 | 2.07 | 2.17 | 0.70 | 0.80 | 15.49 | 17.56 |
| ***SX75*** | 132.75 | 145.27 | 2.56 | 2.36 | 0.79 | 0.75 | 14.18 | 15.92 |
| ***SX77*** | 141.00 | 145.11 | 2.70 | 2.25 | 0.87 | 0.81 | 14.63 | 16.21 |
| ***SX79*** | 135.11 | 120.38 | 2.35 | 2.07 | 0.76 | 0.74 | 14.91 | 12.86 |
| ***SX81*** | 147.43 | 142.55 | 2.94 | 2.05 | 0.90 | 0.70 | 16.90 | 15.19 |
| ***SX83*** | 131.67 | 123.71 | 2.65 | 2.75 | 0.84 | 0.86 | 14.53 | 14.26 |
| ***SX85*** | 148.11 | 150.10 | 2.70 | 2.51 | 0.88 | 0.81 | 16.39 | 16.30 |
| ***SX87*** | 154.75 | 131.23 | 3.09 | 2.11 | 0.92 | 0.71 | 17.23 | 14.35 |
| ***SX89*** | 147.10 | 110.56 | 2.41 | 1.54 | 0.82 | 0.64 | 14.67 | 11.70 |
