# Supplementary material for: Characterization of the olive endophytic community in genotypes displaying a contrasting response to Xylella fastidiosa
Source: BMC Plant Biol. 2024 Apr 25;24:337. doi: 10.1186/s12870-024-04980-2 (PMC11044560; doi:10.1186/s12870-024-04980-2)
Supplement: Supplementary file 7 — Supplementary Material 7 [file 12870_2024_4980_MOESM7_ESM.docx]

**Supplementary Table S6.** **Top 20 results of the RNAseq application** **(DESeq2 method) on SeG samples using the MicrobiomeAnalyst software.** Data were grouped according to genetic features (K1_Ciciulara, K1_Leccino, K1_Others, Not_K). Results indicate differentially abundant features for bacteria (A) and fungi (B) sorted on False Discovery Rate (FDR, cutoff = 0.05). Data in bold are statistically significant. Log_2_FC = log2 fold change; lfcSE = standard error value.

**A**

| **Features** | **Pvalues** | **FDR** | **Log_2_FC** | **lfcSE** |
| --- | --- | --- | --- | --- |
| ***Xylella*** | **2.66e-15** | **1.33e-13** | **25.326** | **32.034** |
| ***Methylobacterium*** | **1,23e-03** | **3,06e-02** | **20.196** | **3.818** |
| ***unkn__Spirulinaceae(f)*** | **4,44E-01** | **0.00074002** | **60.018** | **14.699** |
| ***unkn__Spirulinales(o)*** | **0.00018785** | **0.0023481** | **63.818** | **17.087** |
| ***unkn__Nostocales(o)*** | **0.00036645** | **0.0035525** | **42.448** | **11.913** |
| ***unkn__Aphanizomenonaceae(f)*** | **0.0004263** | **0.0035525** | **52.849** | **1.5** |
| ***unkn__Mollicutes(c)*** | **0.00075366** | **0.0053833** | **80.483** | **23.887** |
| ***unkn__Nostocaceae(f)*** | **0.0017265** | **0.010128** | **40.516** | **12.929** |
| ***unkn__Bacteria(d)*** | **0.001983** | **0.010128** | **22.832** | **0.73823** |
| ***unkn__Clostridia(c)*** | **0.0020256** | **0.010128** | **48.661** | **15.766** |
| ***unkn__Actinobacteria(c)*** | **0.0027509** | **0.011979** | **33.281** | **11.115** |
| ***unkn__Actinobacteria(p)*** | **0.0028751** | **0.011979** | **4.019** | **13.483** |
| ***unkn__Alphaproteobacteria(c)*** | **0.0045646** | **0.017556** | **25.982** | **0.91606** |
| *Halospirulina* | 0.018974 | 0.067766 | 37.047 | 15.791 |
| *Dolichospermum* | 0.023965 | 0.079885 | 30.181 | 13.368 |
| *unkn__Betaproteobacteria(c)* | 0.03571 | 0.11159 | 25.827 | 12.297 |
| *unkn__Firmicutes(p)* | 0.043109 | 0.12679 | 25.328 | 12.522 |
| *unkn__Kineosporiaceae(f)* | 0.056398 | 0.15666 | -55.568 | 29.125 |
| *unkn__Rhizobiales(o)* | 0.061884 | 0.16285 | 41.805 | 2.239 |
| *unkn__Cyanobacteria(p)* | 0.093149 | 0.23287 | 1.544 | 0.91959 |

**B**

| **Features** | **Pvalues** | **FDR** | **Log2FC** | **lfcSE** |
| --- | --- | --- | --- | --- |
| ***Rhodotorula*** | **0.00013782** | **0.019984** | **-54.356** | **14.259** |
| *Preussia* | 0.00085186 | 0.06176 | 6.244 | 18.721 |
| *Neocladosporium* | 0.0024719 | 0.092813 | 34.154 | 11.284 |
| *Ochrocladosporium* | 0.0025604 | 0.092813 | 5.126 | 16.996 |
| *Libertasomyces* | 0.0040094 | 0.11627 | 34.988 | 1.216 |
| *Sporormiella* | 0.011244 | 0.24398 | 86.621 | 3.417 |
| *Diaporthe* | 0.011778 | 0.24398 | 31.839 | 12.641 |
| *Lasiodiplodia* | 0.013539 | 0.24539 | -14.841 | 0.60104 |
| *Mollisia* | 0.018997 | 0.27993 | 70.237 | 29.945 |
| *Phaeosclera* | 0.02122 | 0.27993 | -41.937 | 18.202 |
| *unkn__Teratosphaeriaceae(f)* | 0.022136 | 0.27993 | 25.385 | 11.095 |
| *unkn__Taphrinomycetes(c)* | 0.023166 | 0.27993 | -42.927 | 18.905 |
| *Myriospora* | 0.02924 | 0.2987 | -29.798 | 13.667 |
| *Zasmidium* | 0.030274 | 0.2987 | 6.17 | 28.479 |
| *Acremonium* | 0.0309 | 0.2987 | -28.889 | 13.385 |
| Aureobasidium | 0.03571 | 0.11159 | 25.827 | 12.297 |
| *unkn__Firmicutes(p)* | 0.043109 | 0.12679 | 25.328 | 12.522 |
| *unkn__Kineosporiaceae(f)* | 0.056398 | 0.15666 | -55.568 | 29.125 |
| *unkn__Rhizobiales(o)* | 0.061884 | 0.16285 | 41.805 | 2.239 |
| *unkn__Cyanobacteria(p)* | 0.093149 | 0.23287 | 1.544 | 0.91959 |
