# Supplementary material for: Characterization of the olive endophytic community in genotypes displaying a contrasting response to Xylella fastidiosa
Source: BMC Plant Biol. 2024 Apr 25;24:337. doi: 10.1186/s12870-024-04980-2 (PMC11044560; doi:10.1186/s12870-024-04980-2)
Supplement: Supplementary file 8 — Supplementary Material 8 [file 12870_2024_4980_MOESM8_ESM.docx]

**Supplementary Table S7.** Sample differentiation represented by the *Xylella fastidiosa* concentration (CFU/mL; 0 = 0-100; 1 = 101-10,000; 2 = 10,001-100,000; 3 = 100,001-1,000,000; 4 = 1,000,001-over) based on information obtained by Pavan et al. (2021) [32].

| ***Xf* concentration** | **SeGs** | **CTLs** |
| --- | --- | --- |
| **Group 0**  **(0-100 CFU/mL)** | SX25  SX27  SX30  SX61  SX67  SX75  SX81  SX89 |  |
| **Group 1**  **(101-10,000 CFU/mL)** | SX29  SX63  SX65  SX69  SX77  SX83 | SX36 |
| **Group 2**  **(10,001-100,000 CFU/mL)** | SX71  SX79  SX85  SX87 | SX25  SX29  SX83 |
| **Group 3**  **(100,001-1,000,000 CFU/mL)** | SX31  SX32 | SX32  SX27  SX63  SX65  SX67  SX69  SX73  SX77  SX79  SX85  SX89 |
| **Group 4**  **(1,000,001-over CFU/mL)** | SX73 | SX61  SX75  SX87  SX30  SX31  SX71 |
|  |  |  |
