# Supplementary material for: Characterization of the olive endophytic community in genotypes displaying a contrasting response to Xylella fastidiosa
Source: BMC Plant Biol. 2024 Apr 25;24:337. doi: 10.1186/s12870-024-04980-2 (PMC11044560; doi:10.1186/s12870-024-04980-2)
Supplement: Supplementary file 9 — Supplementary Material 9 [file 12870_2024_4980_MOESM9_ESM.docx]

**Supplementary Table S8.** Classification of SeG samples according to genetic information reported in Pavan et al. [32].

| **K1_Ciciulara^1^** | **K1_Leccino^2^** | **K1_Others^3^** | **Not_K^4^** |
| --- | --- | --- | --- |
| SX25  SX27  SX29 | SX31  SX61  SX65 | SX73  SX75  SX87 | SX30  SX32  SX71 |
|  | SX67 |  | SX69 |
|  | SX77 |  | SX63 |
|  | SX79 |  | SX85 |
|  | SX81 |  |  |
|  | SX83 |  |  |
|  | SX89 |  |  |

^1^K1_Ciciulara= genotypes closely related to the cultivar ‘Ciciulara’

^2^K1_Leccino= genotypes closely related with the cultivar ‘Leccino’

^3^K1_Others= genotypes grouped in K1 but not attributable to a specific cultivar

^4^Not_K= genotypes not falling in any of the above mentioned clusters.
